# Accelular nanofibrous bilayer scaffold intrapenetrated with polydopamine network and implemented into a full-thickness wound of a white-pig model affects inflammation and healing process

**DOI:** 10.1186/s12951-023-01822-5

**Published:** 2023-03-07

**Authors:** Katarína Kacvinská, Veronika Pavliňáková, Petr Poláček, Lenka Michlovská, Veronika Hefka Blahnová, Eva Filová, Martin Knoz, Břetislav Lipový, Jakub Holoubek, Martin Faldyna, Zdeněk Pavlovský, Monika Vícenová, Michaela Cvanová, Jiří Jarkovský, Lucy Vojtová

**Affiliations:** 1 CEITEC – Central European Institute of Technology, Brno University of Technology, Purkyňova 656/123, 612 00 Brno, Czech Republic; 2 Institute of Experimental Medicine of the Czech Academy of Sciences, Vídeňská142 20, 1083 Prague 4, Czech Republic; 3 Department of Burns and Plastic Surgery, Faculty of Medicine, Institution Shared With University Hospital Brno, Masaryk University, Jihlavská, 20, 625 00 Brno, Czech Republic; 4 Department of Plastic and Aesthetic Surgery, Faculty of Medicine, St. Anne’s University Hospital, Masaryk University, Pekařská, 664/53, 602 00 Brno, Czech Republic; 5 Veterinary Research Institute, Hudcova 296/70, 621 00 Brno, Czech Republic; 6 Institute of Pathology, Faculty of Medicine, University Hospital Brno, Masaryk University, Brno, 625 00 Czech Republic; 7 Institute of Biostatistics and Analyses, Faculty of Medicine, Masaryk University, Kamenice 5, 625 00 Brno, Czech Republic

**Keywords:** Bilayer, Chitosan, Collagen, Oxidized cellulose, Polydopamine, Wound healing

## Abstract

Treatment of complete loss of skin thickness requires expensive cellular materials and limited skin grafts used as temporary coverage. This paper presents an acellular bilayer scaffold modified with polydopamine (PDA), which is designed to mimic a missing dermis and a basement membrane (BM). The alternate dermis is made from freeze-dried collagen and chitosan (Coll/Chit) or collagen and a calcium salt of oxidized cellulose (Coll/CaOC). Alternate BM is made from electrospun gelatin (Gel), polycaprolactone (PCL), and CaOC. Morphological and mechanical analyzes have shown that PDA significantly improved the elasticity and strength of collagen microfibrils, which favorably affected swelling capacity and porosity. PDA significantly supported and maintained metabolic activity, proliferation, and viability of the murine fibroblast cell lines. The in vivo experiment carried out in a domestic Large white pig model resulted in the expression of pro-inflammatory cytokines in the first 1–2 weeks, giving the idea that PDA and/or CaOC trigger the early stages of inflammation. Otherwise, in later stages, PDA caused a reduction in inflammation with the expression of the anti-inflammatory molecule IL10 and the transforming growth factor β (TGFβ1), which could support the formation of fibroblasts. Similarities in treatment with native porcine skin suggested that the bilayer can be used as an implant for full-thickness skin wounds and thus eliminate the use of skin grafts.

## Background

Wound healing is an integrated and complex process that begins immediately after injury and involves the release of a large number of regulatory molecules, including pro-inflammatory cytokines, growth factors, and low molecular weight compounds from the serum of injured blood vessels and degranulated platelets [1, 2]. The epidermal defect itself is called a superficial wound, a defect in the epidermis and dermis together with damage to blood vessels, sweat glands, etc. is called a partial thickness wound, while damage to the subcutaneous fat layer is called a full thickness wound and it leads to extensive loss of skin, hair follicles, and glands [3]. There are many studies that are applied with extensive research to the treatment of partial and full-thickness wounds using different materials made of porous foams, hydrogels, or nanofibrous layers of synthetic materials (poly(ethylene glycol) (PEG), poly(ε-caprolactone) (PCL), poly(lactic-co-glycolic acid) (PLGA), poly(lactic acid) (PLA), poly(vinyl alcohol) (PVA), polyurethane films, or silk fibroin) [4–6] and/or natural materials based on collagen (Coll), gelatin (Gel), cellulose, alginate, chitosan, hyaluronan, fibrin or fucoidan materials [7–9].

Coll has been widely used in many applications because the naturally occurring protein consists of three α-domains (polypeptide chains) that provide the main mechanical support for cell attachment and has excellent biocompatibility and biodegradability [10–14]. However, Coll-based scaffolds face rapid biodegradation rates and low mechanical strength. Chemical cross-linking is the most effective strategy to promote stability. Carbodiimides have been widely investigated as suitable crosslinkers for collagen scaffolds [15–17]. Another possibility is the combination of Coll with natural and/or synthetic materials, which brings new functional possibilities to tissue engineering applications [18]. Chitosan (Chit) is a biodegradable, non-toxic, and antibacterial material with a homeostatic effect. Chit is often used in combination with Coll; it accelerates fibroblast formation and enhances early phase reactions related to healing [19, 20]. In our previous studies, [21, 22] we evaluated Coll/Chit scaffolds enriched with fibroblast growth factor 2 (FGF 2) and further in combination with selenium nanoparticles (SeNPs). The results showed support for fibroblast attachment and metabolic activity. In addition, the scaffolds exhibited antibacterial activity against three strains of bacteria, *Escherichia Coli (E. coli)*, *Staphylococcus aureus (S. aureus)*, and methicillin-resistant *S. aureus* (MRSA). Chit can be processed in various forms such as films, hydrogels, fibers, powders, and micro/nanoparticles used in skin tissue engineering [23, 24]. Oxidized cellulose is a biodegradable polymer, with non-immunogenicity, and it promotes the healing of chronic wounds [25, 26]. In combination with Coll, it reduces pro-inflammatory interleukins, reactive oxygen species, and binds to metal ions with increasing concentrations of growth factors and proteinase inhibitors [27]. The addition of calcium salt of oxidized cellulose (CaOC) to electrospun nanofibers provided a unique inhibitory effect on *E. coli* bacteria [28]. Poly(ε-caprolactone) (PCL) is a synthetic, biocompatible, linear aliphatic polyester that is hydrophobic, it degrades relatively slowly, and has good mechanical properties. PCL scaffolds have been used as in vivo tissue implants for various medical applications and have shown great potential for wound healing, bone tissue engineering, cardiovascular tissue engineering, and nerve regeneration [29, 30]. Dopamine is a molecule that forms natural adhesion between the material surfaces or sticks small molecules. It is synthesized in the body by cells and has an amino acid sequence similar to that of mussel protein, which has the ability to bind to many surfaces in an aqueous environment [31]. An attractive property of dopamine is its auto-polymerization, which has been reported to occur in Tris buffer with pH of 8.5, where dopamine leads to polydopamine (PDA) films and nanofibers [32–34].

In recent years, studies based on multilayer scaffolds in the treatment of full-thickness wounds have advantages over a single layer dressing because they can functionally replace both dermal and epidermal components. Acellular bilayer materials were prepared by a combination of natural and synthetic materials, e.g., Chit/PCL nanofibrous mats, PLLA-microporous disc [35]. Furthermore, the Coll/Chit scaffold enriched with recombinant human vascular endothelial growth factor (rhVEGF) and antibacterial gentamicin were encapsulated in PLGA microspheres [36]. A trilayer Chit-based scaffold was prepared to more accurately replicate full-thickness skin striation than a single or bilayer scaffold, which required weeks of co-culture of fibroblasts and keratinocytes to achieve similar striation [37]. There are many other existing studies that consider the potential use of acellular multilayered scaffolds, not only in skin tissue, [38–40] but also in vascular tissue [41] and bone tissue engineering [42].

This study aims to develop an acellular PDA-modified bilayer scaffold and to enhance mechanical and biological support in full-thickness porcine skin wound reconstruction. The bilayer is made of porous Coll/polysaccharide foam (Chit or CaOC) with the aim of mimicking a dermis-like structure and a basal membrane-like structure, characterized by a nanofibrous layer made of biocompatible polymers gelatin, PCL, and CaOC. The PDA-modified bilayer significantly changes mechanical properties and promotes stability, leading to different water absorption and material morphology. These changes also allowed cells to proliferate with maintained viability. In vitro evaluation of cytotoxicity and metabolic and proliferation activity of murine fibroblasts demonstrated a non-cytotoxic effect of implanted bilayers. In this study, the healing process was monitored for a longer period of time, as well as the effect of PDA after transplantation, which shows the histological analysis of inflammatory and anti-inflammatory cytokines. PDA can enhance the inflammatory phase at the beginning of wound healing and shows possible support for the expansion of growth factor and anti-inflammatory cytokines in the middle and later stages of wound healing compared to native porcine skin treatment.

## Methods

### Materials and chemicals

Bovine Collagen type I, 8 wt.% aqueous solution (Coll, Collado s.r.o., Brno, Czech Republic), chitosan from shrimp shells, 70% DDA, low viscosity (Chit, Sigma-Aldrich, Darmstadt, Germany), calcium salt of oxidized cellulose–degree of oxidation 16–24% and *M*_n_ = 350 kg/mol (CaOC, Synthesia, Pardubice, Czech Republic), acetic acid (99%, Penta s.r.o, Chrudim, Czech Republic), poly(ε-caprolactone) (PCL, 80 kg/mol), gelatin (Gel, Type B, Bioreagent, powder from bovine skin),* N*-(3-Dimethylaminopropyl)-*N*´-ethylcarbodiimide hydrochloride (EDC), *N*-hydroxysuccinimide (NHS), 98% dopamine hydrochloride, tris (hydroxymethyl) aminomethane hydrochloride, ethanol p.a. 99.8%, sodium phosphate dibasic for molecular biology (≥ 98,5%), sodium chloride, calcium chloride, sodium phosphate dibasic dodecahydrate (Na_2_HPO_4_ ·12H_2_O), potassium dihydrogen phosphate (KH_2_PO_4_), potassium chloride (KCl), collagenase from *Clostridium histolyticum*, lysozyme human, the murine fibroblast cell lines 3T3-A31, Dulbecco's modified eagle medium DMEM (D6429), fetal bovine serum FBS (F7524), 2′,7′-bis (2-carboxyethyl)-5(6)-carboxyfluorescein acetoxymethyl ester (BCECF-AM), propidium iodide (P4864), (all from Sigma Aldrich, Darmstadt, Germany), penicillin/streptomycin (15140–122) and DiOC6(3) (D273), (Life Technologies, Eugene, OR, USA), octenidine solution (Octenisept®, Schülke, Germany), Butomidor^®^ inj. (butorphanol tartrate, Vétoquinol, Czech republic), Domitor^®^, Medetomidine, Orion corporation, Finland) Propofol^®^ (Propofolum 1%, Fresenius Kabi Deutschland, Bad Homburg, Germany), Metacam^®^ (meloxicam, Boehringer Ingelheim Vetmedica, Ingelheim/Rhein, Germany), Enroxil^®^ (Enrofloxacin, Krka, Novo mesto, Slovenia) Betadine^®^, (2.5% solution of povidone iodine, EGIS Pharmaceuticals PLC, Budapest, Hungary) were used as received without further purification.

### Preparation of samples

#### Preparation of porous foams and cross-linked bilayers

Porous foams were prepared according to previous work [43]. Briefly, the calculated amount of Coll (0.5 wt.%) and suitable polysaccharide (0.5 wt.%) in the weight ratio of 1:1 was slowly homogenized. Material suspensions were freeze-dried on an Epsilon 2-10D machine (Martin Christ, Osterode am Hartz, Germany). A fibrous layer was electrospun on the surface of the lyophilized porous foam according to [28] modified with the addition of PCL. Nanofibers were prepared as follows: the Gel/PCL/CaOC 70/30/10 polymer solution was prepared in concentrated glacial acetic acid and stirred overnight. Electrospinning was performed using a laboratory Nanospider NS LAB 500 machine (Department of Physical Electronics, Masaryk University, Brno, Czech Republic). The setting parameters were as follows: flow rate of 25 mm‧min^−1^, applied voltage of 60 kV, the distance between the spinning and collecting electrode was set to 15 cm, and the spinning electrode was rotated at a speed of 5 rpm. The ambient conditions were 23 °C, 980 kPa, and 40% humidity. A cross-linking agent of the carbodiimide system in ethanol (EDC/NHS in a molar ratio 2/1) was used to cross-link the porous foam and the nanofibrous layer. After 2 h of the cross-linking process, the bilayer was washed twice with 0.1 M Na_2_HPO_4_ followed three times with ultrapure water to remove by-products. Subsequently, the bilayers were freeze-dried and stored in desiccators prior use.

#### Preparation of PDA-coated cross-linked bilayers

Porous foams and nanofibrous layers were prepared as described in *3.2.1*. Briefly, a nanofibrous layer was electrospun on the freeze-dried porous foam and both parts were cross-linked and washed after 2 h. A solution of dopamine hydrochloride (2 mg.mL^−1^ in 0.01 M Tris HCl) was prepared before the second freeze-drying process. Tris HCl was used as an initiator for dopamine polymerization, resulting in black polydopamine (PDA) [31]. Cross-linked bilayers were immersed in a solution of dopamine hydrochloride and maintained for another 24 h under aerobic conditions. The PDA-intrapenetrated samples were then washed 5 times in water to remove residual unbound dopamine, and subsequently the samples were lyophilized again. Samples were always prepared either in a volume of 500 μL in 24-well plates for *in vitro* testing (only porous foams), or in a volume of 80 mL in 12 × 12 cm square plastic plates for biomechanical evaluation (bilayers). All types of prepared samples are summarized in Table 1. An explanation of sample abbreviations is as follows: The letter *N* indicates the presence of a cross-linked nanofibrous layer (e.g., the abbreviation of Coll/Chit-N/PDA belongs to the bilayer formed by Coll/Chit foam with cross-linked nanofibers, coated with PDA). The letters *NX* indicate the presence of a non-cross-linked nanofibrous layer, as it can be seen in Table 1.

**Table 1:** Summary of prepared samples

**Porous foam composition**	**Abbreviation of foam**
Collagen foam	Coll
Collagen/Chitosan foam	Coll/Chit
Collagen/CaOC foam	Coll/CaOC
**Porous foam coated with PDA**	**Abbreviation of foam**
Collagen foam coated with PDA	Coll/PDA
Collagen/Chitosan coated with PDA	Coll/Chit/PDA
Collagen/CaOC coated with PDA	Coll/CaOC/PDA
Non-cross-linked bilayers*	**Abbreviation of bilayer**
Collagen bilayer (foam + nanofibers)	Coll-NX
Collagen/Chitosan bilayer (foam + nanofibers)	Coll/Chit-NX
Collagen/CaOC bilayer (foam + nanofibers)	Coll/CaOC-NX
**Cross-linked bilayers**	**Abbreviation of bilayer**
Collagen bilayer (foam + nanofibers)	Coll-N
Collagen/Chitosan bilayer (foam + nanofibers)	Coll/Chit-N
Collagen/CaOC bilayer (foam + nanofibers)	Coll/CaOC-N
**Cross-linked bilayers coated with PDA**	**Abbreviation of bilayer**
Collagen bilayer (foam + nanofibers and PDA coating)	Coll-N/PDA
Collagen/Chitosan bilayer (foam + nanofibers and PDA coating)	Coll/Chit-N/PDA
Collagen/CaOC bilayer (foam + nanofibers and PDA coating)	Coll/CaOC-N/PDA

^*^ All non-cross-linked bilayers are excluded from following experiments due to the low mechanical properties and are mentioned only in SEM observation

### Sample characterization

#### Structure and morphology

A scanning electron microscope MIRA3 (TESCAN, Brno, Czech Republic) was used to study the morphology and adhesion of the prepared bilayers. Images were taken in a secondary electron emission mode, the scan mode was DEPTH, the beam density was 10 and the high voltage was 10 kV. The working distance was set to 15 mm. The surface of the samples was coated with a 20 nm thin layer of Au/Pd using EM ACE 600 (Leica Microsystems, Wetzlar, Germany). The pore size was characterized from SEM images using ImageJ software and SEM Image Pore Extractor (SEMIPE). A minimum of five and a maximum of ten images with the same resolution were taken from each sample. From each image, 40–90 pores were measured. Data were evaluated using a 2-sample T-test, which assumes unequal variances and unequal sample sizes. The level of significance was set at *p < 0.05 **p < 0.01and ***p < 0.001.

#### Fourier-transformed infrared study results

A Fourier-transformed infrared spectroscopy (FTIR) with attenuated total reflectance (ATR-FTIR, Vertex 70/70v, Bruker, Billerica, MA, USA) was performed to characterize material composition of porous foam with and without PDA and material composition of the PDA bilayers. Presented ATR-FTIR spectra were taken from averaging 32 scans with a spectral resolution of 2 cm^−1^. The displayed spectra in the wavenumber range of 4000–500 cm^−1^ were normalized using min–max normalization (OPUS software, Bruker, Billerica, MA, USA). The ATR-FTIR spectra were measured under evacuated conditions from all samples, each placed on a diamond ATR crystal.

#### Dynamic mechanical analysis

An RSA G2 dynamic mechanical analyzer (TA Instruments Inc., New Castle, USA) was used to measure the tensile properties of prepared bilayers. The prepared samples were cut into strips with a length of 40 mm and a width of 10 mm. Thickness varies with the type of sample (0.20 mm–0.60 mm) and samples were measured with Digital Caliper 0–150 mm (Groningen, Netherlands). The first biomechanical tests were performed at room temperature at 23 °C and the second test involved constant hydration conditions with phosphate buffer at 36 °C in a built chamber surrounding the grip. Before each measurement, 10 min of swelling was allowed for each sample to swell the bilayers. The relationship between stress and strain is shown along with the corresponding elastic modulus*.* Data analysis using Microsoft Excel was used for the statistical evaluation of five samples of the same bilayer. Data were evaluated using a 2-sample T-test, which assumes unequal variances and unequal sample sizes. The level of significance was set at ** p < 0.01and ***p < 0.001.

#### Swelling capacity

The bilayers were cut into 1 × 2 cm strips and immersed in a water solution to test their hydrolytic stability under ambient conditions. Each sample was weighted before immersion (*Wi*). The weight of swollen samples (*Ws*) was also recorded after gently removing the surface water with filter paper at several intervals: 1, 2, 5, 10, 15, 20, 30, 45, 60, 90, 120, 150, and 180 min. A swelling ratio was calculated to define the exact amount of swelling caused by water absorption, and the swelling curve was obtained. The swelling ratio was calculated according to Eq (1)

1 $$Swelling\;\,Ratio = \frac{{W_{s} }}{{W_{i} }}$$

The samples were measured in triplicates and the results are shown as mean ± standard deviation.

#### Enzymatic stability

Collagenase from *Clostridium histolyticum* was used to investigate in vitro degradation studies of chemically cross-linked bilayers. Degradation was carried out in prepared phosphate buffered saline (PBS) at physiological pH of 7.4 at 37 °C. After one hour of swelling, samples were removed from the PBS, subsequently weighted, and placed in the collagenase solution (c = 2.2 mg∙L^−1^). After every 2, 4, 8, 24, 48, 72, 96, 120 and 144 h, excess PBS was blotted onto the filter paper, following the weight notation, as well as the percentage of weight loss calculation using Eq. (2)

2 $$Weight\;Loss = 100 - \left( {\frac{{W_{i} \cdot 100}}{{W_{s} }}} \right)\;[\% ]$$

where *Ws* represents the weight of the scaffold after 1 h of swelling and *Wi* represents the weight of the digested scaffold. Three measurements of each type of sample were recorded and shown as mean ± standard deviation.

#### In vitro* cytotoxicity assessment*

Murine fibroblast cell lines 3T3-A31 were cultured in culture medium containing DMEM (high glucose, D6429, Sigma-Aldrich, St. Louis, MO, USA), 10% FBS, and 1% penicillin/streptomycin. 70,000 cells/scaffold (with a diameter of 10 mm and a height of 4–5 mm) were seeded for a period of 14 days.

Metabolic activity was determined by the CellTiter 96^®^ Aqueous One Solution Cell Proliferation (MTS) metabolic assay (CellTiter 96^®^ Aqueous One Solution Cell Proliferation Assay, Promega corp., Madison, WI, USA), where the MTS tetrazolium compound was added directly to the cell culture medium in a 1:5 ratio. Metabolically active cells reduced the MTS reagent and generated a colored formazan dye that is soluble in cell culture medium. Formazan dye was quantified by measuring the absorbance at 490 nm, reference 690 nm using Tecan Infinite M200 Pro. The samples were carried out in biological quadruplicates; the results are shown as mean ± standard deviation.

The Quant-iT™ dsDNA Assay Reagent (Invitrogen) assay determined cell proliferation, as it quantified the amount of double-stranded DNA. The assay contains a fluorescent dye activated once it is bound to dsDNA. Fluorescence was measured at λex = 485 nm and λem = 523 nm. The samples were carried out in biological quadruplicates; the results are shown as mean ± standard deviation.

Cell viability was assessed by live-dead staining of three samples. 2′,7′-bis (2-carboxyethyl)-5(6)-carboxyfluorescein acetoxymethyl ester (BCECF, Sigma-Aldrich, Saint Luis, MO, USA) was used to visualize the membranes of living cells and propidium iodide to visualize the nuclei of dead cells. Samples were observed using a Zeiss LSM 880 Airyscan confocal microscope. Excitation/emission was set as follows: BCECF λex = 488 nm/λem = 505–545 nm, PI λex = 560 nm/λem ˃ 575 nm.

The cell distribution on the scaffold and the morphology were observed using 3,3'-Dihexyloxacarbocyanine Iodide (Invitrogen™) DiOC6(3)/propidium iodide (ThermoFisher Scientific™) staining. Excitation/emission was set as follows: DiOC6(3) λex = 488 nm/λem = 505–545 nm, PI λex = 560 nm/λem ˃ 575 nm. The signal from the nuclei was further used to determine the depth of penetration of the cell into the scaffold.

The statistical significance was performed using one-way analysis of variance (ANOVA) in Sigma Stat software 3.5 (Systat Software, California, USA).

#### In vivo* experiment*

The *in* *vivo* experimental part was performed in a total of 2 female pigs (*Sus Scrofa domesticus*) of the production hybrid line with an initial weight of 70 ± 5 kg. All phases of the experiment lasted 6 months. The pigs were supplied by a local production company approved by the Ministry of Agriculture of the Czech Republic and housed at the Veterinary Research Institute (Brno, Czech Republic) in experimental stables certified by the Ministry of Agriculture of the Czech Republic. The study was carried out according to the Declaration of Helsinki and was approved by the Institutional Review Board of the Veterinary Research Institute (protocol code 12/2016 with approval from 21 April 2016) and by the Branch Commission for Animal Welfare of the Ministry of Agriculture of the Czech Republic (permission number 34715/2016-MZE-17214 from 15 June 2016). Upon arrival at the research facility, the pigs were housed for two weeks prior to the experimental procedure challenge and housed individually in stainless-steel cages located in isolated rooms with controlled regime and independent ventilation. Rooms were kept at a temperature of 21 °C, a relative humidity in the range of 40–60%, and a ventilation of approximately 15 air changes per hour.

**Fig. 1:**
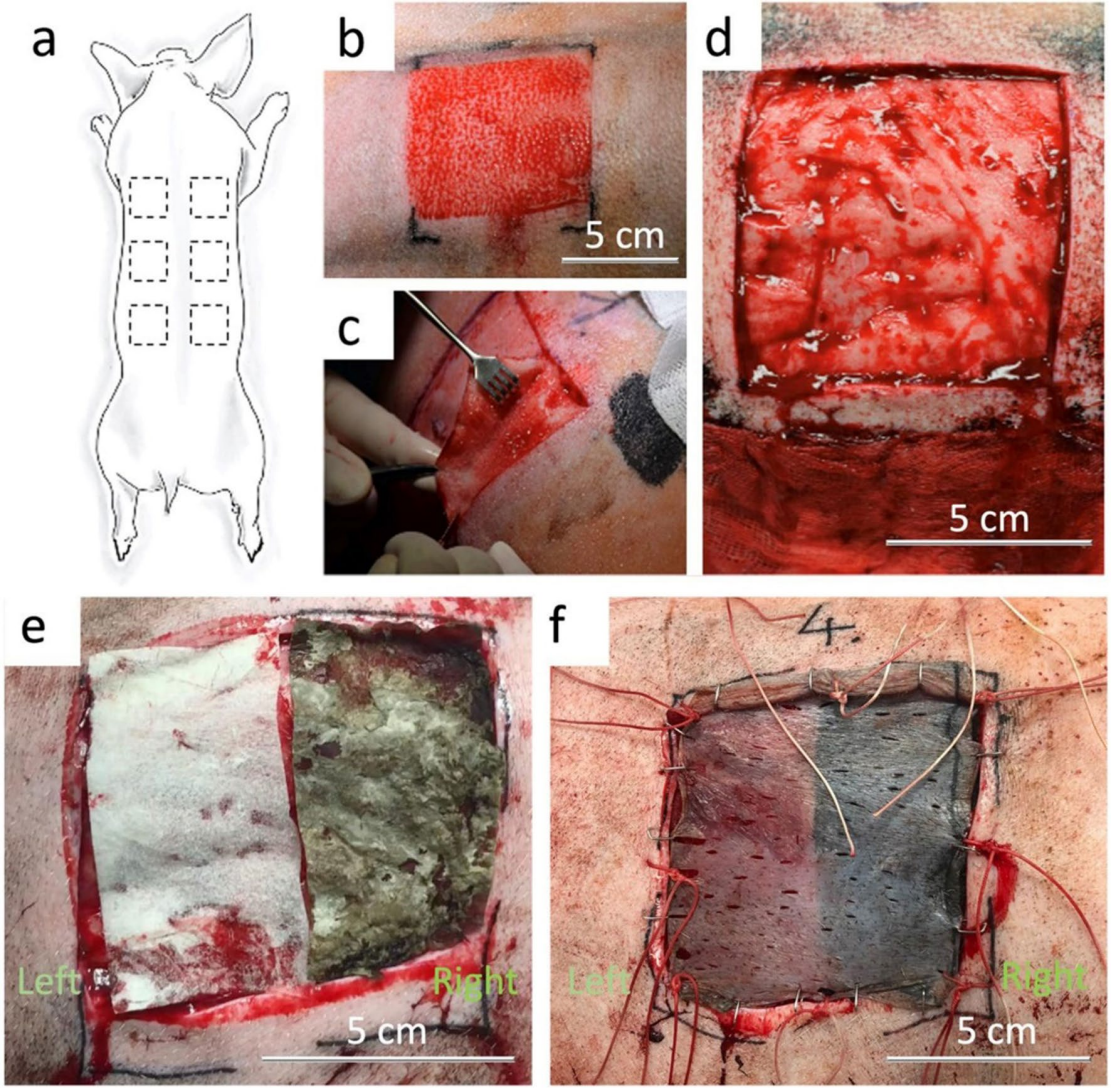
Creation of 6 full-thickness skin wounds 8 × 8 cm **a** scheme of wounds location, **b** split-thickness skin graft (STSG) donor site, **c** full-thickness excision, **d** wound bed preparation prior to scaffold application, **e** bilayer scaffold made of collagen/oxidized cellulose foam with cross-linked nanofibers—Coll/CaOC-N (left) and bilayer scaffold coated with polydopamine—Coll/CaOC-N/PDA (right) application directly to wound bed, **f** application of STSG on bilayer scaffold, covering the full-thickness skin defect in one-step procedure

All surgical procedures were performed under general anesthesia. Pre-medication and analgesia during the surgery were performed by Butomidor inj. (butorphanol tartrate) at a dose of 0.1 mg‧kg^−1^ b.w. s.c. Anesthesia was performed with Medetomidine at a dose of 0.5 mg‧kg^−1^ body weight. Furthermore, general anesthesia was maintained throughout the surgery by continuous administration of Propofol 1% at a dose (8–15 mg‧kg^−1^ b.w. i.v.). Immediately after surgery, the analgesic Metacam (meloxicam) was used at a dose of 0.1 mg‧kg^-1^ b.w. s.c. once a day for three consecutive days. Experimental animals received systematic antibiotic therapy (Enrofloxacin 15 mg‧kg^-1^ b.w. once daily i.m. for 10 days). After shaving the fur off the back of the pig, antisepsis of the donor area was performed with a 2.5% solution of povidone iodine. Subsequently, using an electrodermatome (Zimmer Biomet^®^ Air Dermatome, Zimmer Biomet, Indiana, USA), a 0.20 mm thin split- thickness skin graft (STSG) was removed with an area of 8 × 8 cm in square at 6 sites of planned skin defects. A sharp excision of an area measuring 8 × 8 cm of full-thickness skin (2–2.3 cm depth) was performed at the site of the removed skin grafts (Fig. 1). This was followed by the application and fixation of the nanostructured scaffold and its STSG cover. The nanostructured scaffold consists of Coll and CaOC-based foam as the dermis layer (thickness around 2 mm) and the nanofibers layer as a basal membrane (thickness around 200 µm). The scaffold on the right was PDA-coated (Fig. 1e) and on the left was without PDA. An epidermal graft without scaffold was used as a control. All implants were fixed to the wound using a skin stapler (Single-use Skin Stapler B. Braun^®^, B. Braun, Germany), covered with greasy tulle and mule moistened with Octenidine solution, and secured with a pressure bandage. Microsoft Excel and its = RAND() function were used to randomize the types of biomaterials used in individual full-thickness skin defects.

The first dressing change followed on the seventh postoperative day, when the viability of the graft was verified. The defects were then tied with a wet mule and a greasy tulle. Histological and immunohistological samples were taken under general anesthesia at individual stages of defect healing on the 7th and 14th postoperative day and then in the 3rd and 6th postoperative month. The post-incision defect closure was performed by direct absorbable suture.

#### Histological analysis and qPCR analysis of tissue samples

Formaldehyde-fixed, paraffin-embedded tissue samples were processed for 2 histological sections per sample and stained with hematoxilin and eosin. Light microscopy was used to evaluate the histological images. For performance, mRNA was stabilized in tissue samples with an RNA Later kit (Quiagen, The Netherlands). Total RNA was isolated using an RNeasy Mini Kit (Quiagen, The Netherlands) from 4 samples per group and reverse transcribed with the oligo-dT primer and MMLV (Invitrogen, USA) reverse transcriptase. Primers for all genes (e.g., IL1β, TNFα, TGFβ1, IL10) and the reference gene (HPRT) were used in previous publications of the team [44–46]. Based on the results obtained from histological analyzes, other genes (examination of genes associated with cell death) were also considered. For RT-PCR, a Light Cycler 480 (Roche, Switzerland) was used. Each PCR reaction consisted of QuantiTect Sybr Green master mix (Quiagen, The Netherlands), 1 μM of each primer and 1.0 μL of cDNA in a total volume of 10 μL. Each sample was run in duplicate. The expression of a particular gene was calculated as a multiple of the expression of the reference gene using the following formula: [1/(2Ct GOI)]/[1/(2CtHPRT)].

The mean expression of the HRT unit of pro-inflammatory, anti-inflammatory cytokines and growth factors was compared using the T-test. Immunohistological samples with the Coll/CaOC, samples with the addition of PDA and control group were compared in each time point separately.

## Results

### PDA influences the structure and morphology of the bilayer

**Fig. 2:**
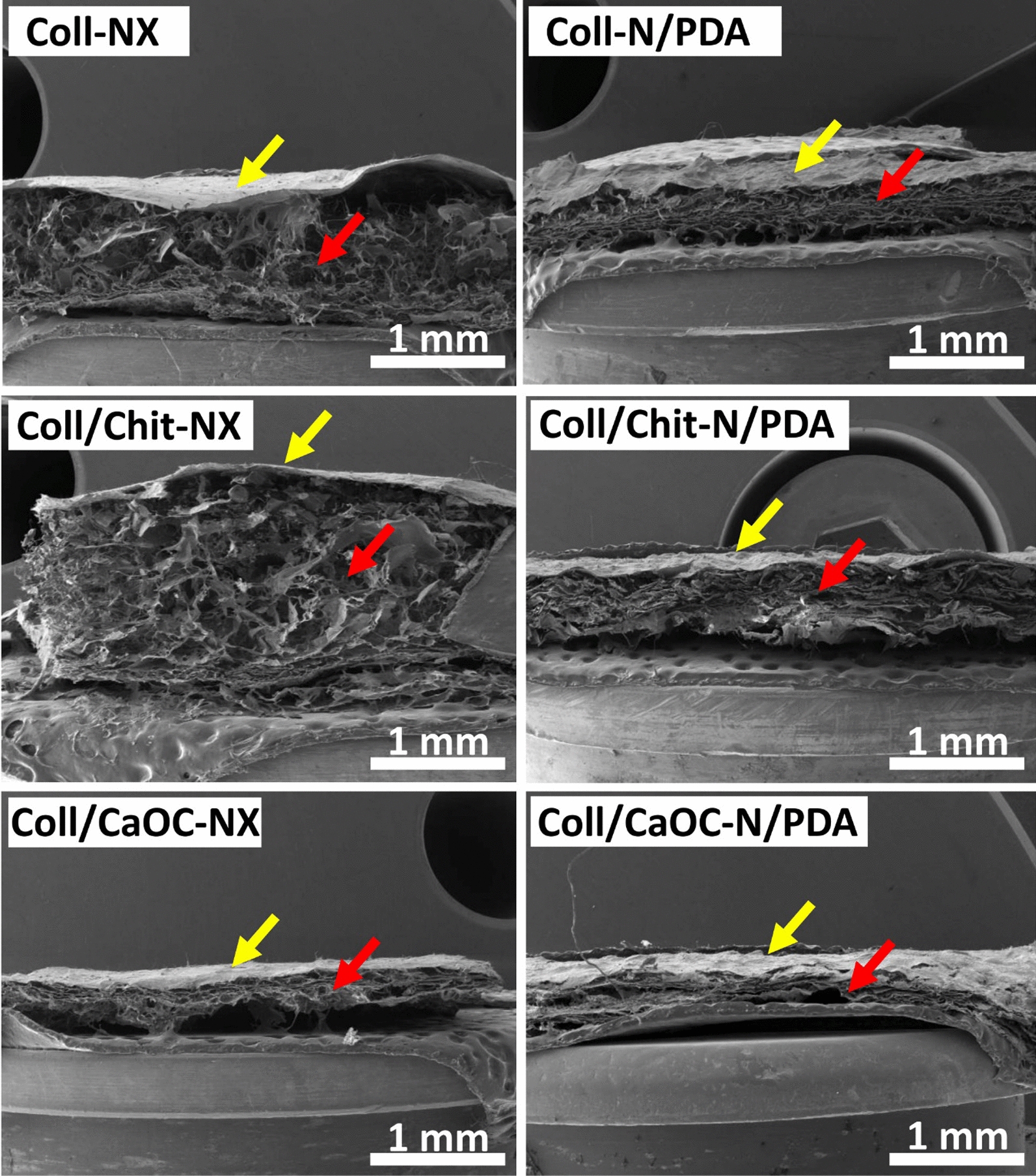
SEM visualization of bilayers made of porous foam and nanofibers. Collagen foam with cross-linked nanofibers is represented as Coll-N; collagen/chitosan foam with cross-linked nanofibers is represented as Coll/Chit-N; and collagen/oxidized cellulose foam with cross-linked nanofibers is represented as Coll/CaOC-N. The next mark ‘NX’ represents the non-cross-linked bilayer. The mark ‘PDA’ represents the polydopamine coating on cross-linked bilayers. The nanofibrous layer is placed on the surface of the sample (yellow arrow). A porous scaffold structure can be seen below it (red arrow)

**Fig. 3:**
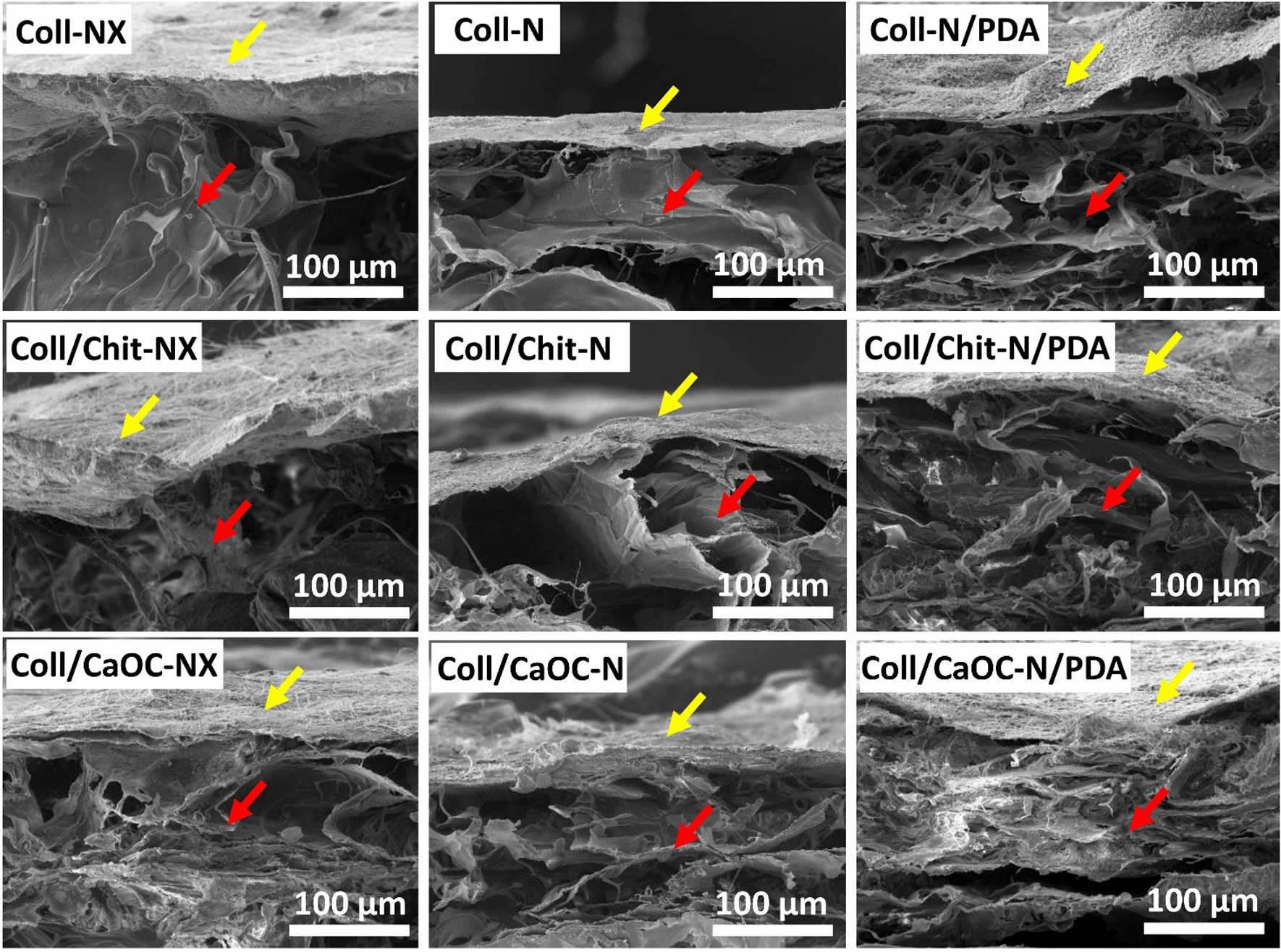
Detailed SEM visualization of adhesion between porous foam and nanofibers. Collagen foam with cross-linked nanofibers is represented as Coll-N; collagen/chitosan foam with cross-linked nanofibers is represented as Coll/Chit-N; and collagen/oxidized cellulose foam with cross-linked nanofibers is represented as Coll/CaOC-N. The next mark ‘NX’ represents the non-cross-linked bilayer. The mark ‘PDA’ represents the polydopamine coating on cross-linked bilayers. The nanofibrous layer is placed on the surface of the sample (yellow arrow). A porous scaffold structure can be seen below it (red arrow)

**Fig. 4:**
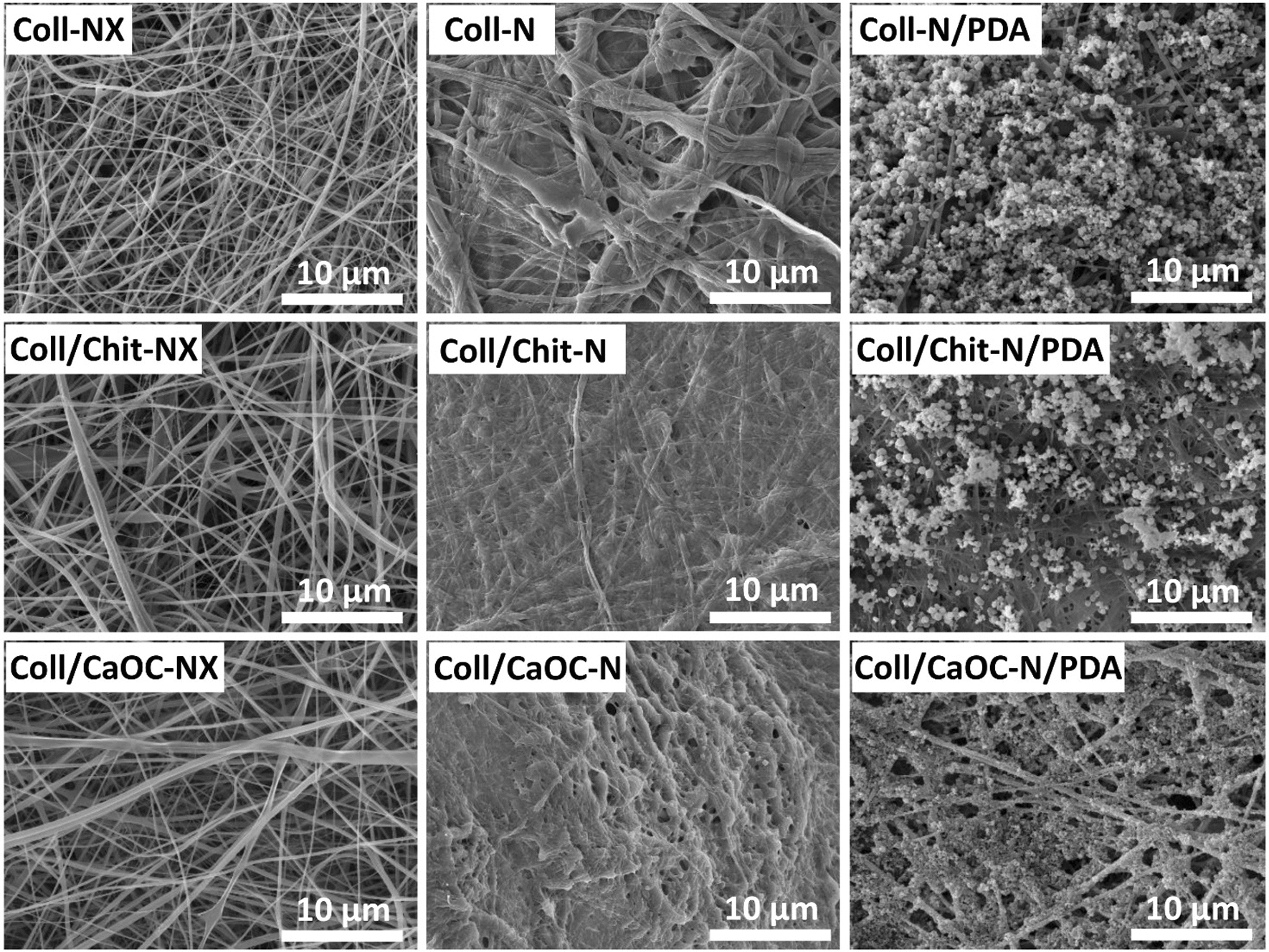
SEM visualization of surface of nanofibrous layers. Collagen foam with cross-linked nanofibers is represented as Coll-N; collagen/chitosan foam with cross-linked nanofibers is represented as Coll/Chit-N; and collagen/oxidized cellulose foam with cross-linked nanofibers is represented as Coll/CaOC-N. The next mark ‘NX’ represents the non-cross-linked bilayer. The mark ‘PDA’ represents the polydopamine coating on cross-linked bilayers

In this study, cross-linked bilayers and PDA-coated cross-linked bilayers were prepared and morphologically compared with non-cross-linked bilayers. Figure 2 shows SEM visualizations of non-cross-linked bilayers and PDA-coated cross-linked bilayers to show the significant effect of both interventions cross-linking and PDA coating. The nanofibrous layer is placed on the surface of the sample (yellow arrow). A porous scaffold structure can be seen below it (red arrow). Figure 3 shows a detailed SEM visualization of the adhesion between the nanofibrous layer and the porous foam. Figure 4 shows the nanofibrous structure of the bilayer. Non-cross-linked nanofibers are smooth with random fiber orientation and with fiber diameter in the range of 370–500 nm (Fig. 4(NX)). Cross-linked nanofibers partially lost their fibrous structure, and the fibers are already firmly attached to each other, exhibiting a more uniform structure (Fig. 4(N)). Non-covalent self-assembly of dopamine in cross-linking bilayers produced PDA precipitates deposited on nanofibers and almost continuously covered the entire fibrous area (Fig. 4(N/PDA)). A slight diversity was observed only for the Coll/CaOC-N/PDA sample. In this case, PDA formed a continuous film on the surface of the bilayer.

**Fig. 5:**
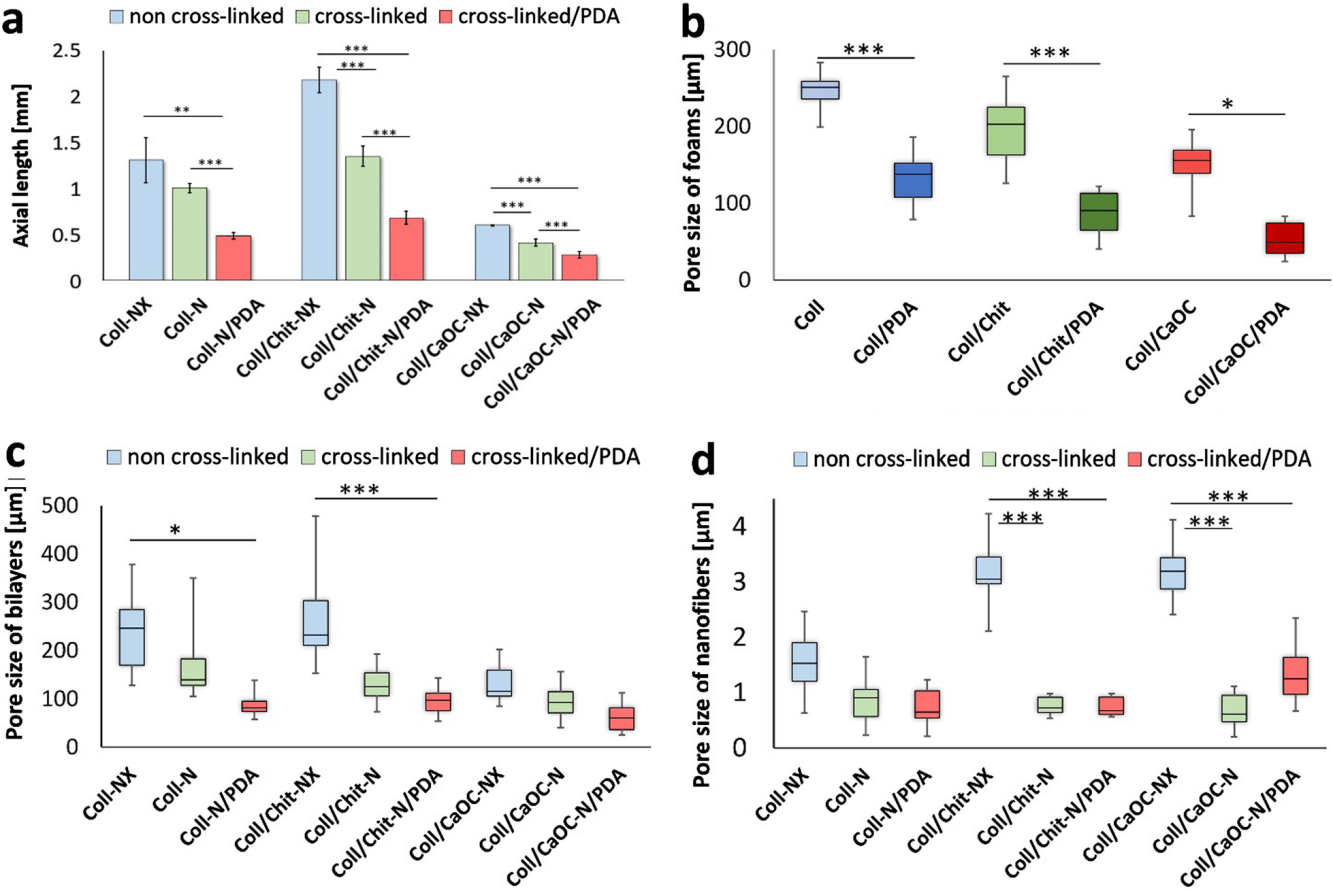
Effect of cross-linking and PDA coating on the bilayer thickness **a**; effect of PDA coating on the pore size of porous foams **b**; effect of cross-linking and PDA coating on the pore sizes of foams measured from the cross-sectional area of bilayers **c**, and pore sizes of nanofibers **d**. Collagen foam with cross-linked nanofibers is represented as Coll-N; collagen/chitosan foam with cross-linked nanofibers is represented as Coll/Chit-N; and collagen/oxidized cellulose foam with cross-linked nanofibers is represented as Coll/CaOC-N. The next mark ‘NX’ represents the non-cross-linked bilayer. The mark ‘PDA’ represents the polydopamine coating on cross-linked bilayers

Figure 5a–d presents the effect of PDA and cross-linking on total bilayer thickness and pore sizes. Here, it is evident that the cross-linking process as well as the PDA addition lead to a decrease in the bilayer thickness; the range of thickness is between 0.3 and 2.2 mm (Fig. 5a). The thickness of these samples was in most cases half that of the original non-cross-linked bilayer. Figure 5b shows the pore sizes of porous foams without the presence of nanofibers, where the pore sizes are in the range of 50–250 µm and where PDA significantly decreases the pore sizes of all foams. The pore sizes of the porous foams were also measured from the cross-sectional area of the bilayers Fig. 5c. A decrease in thickness is followed by a decrease in the pore sizes, while the pores change shape to a thin ellipsoid. Here, there is no significance after cross-linking, only PDA presents significant results in pore reduction in some bilayers. The pore size of the bilayers was reduced from 250 to 80 µm for Coll-NX/PDA and Coll-N/PDA, from 230 to 100 µm for Coll/Chit-NX/PDA and Coll/Chit-N/PDA and from 120 to 60 µm for Coll/CaOC-NX/PDA and Coll/CaOC-N/PDA, respectively. The pore sizes on the surface of the nanofibers are in the range of 0.7–4 µm, influenced by PDA and/or cross-linking (Fig. 5d).

### PDA influences the structure integrity of collagen

**Fig. 6:**
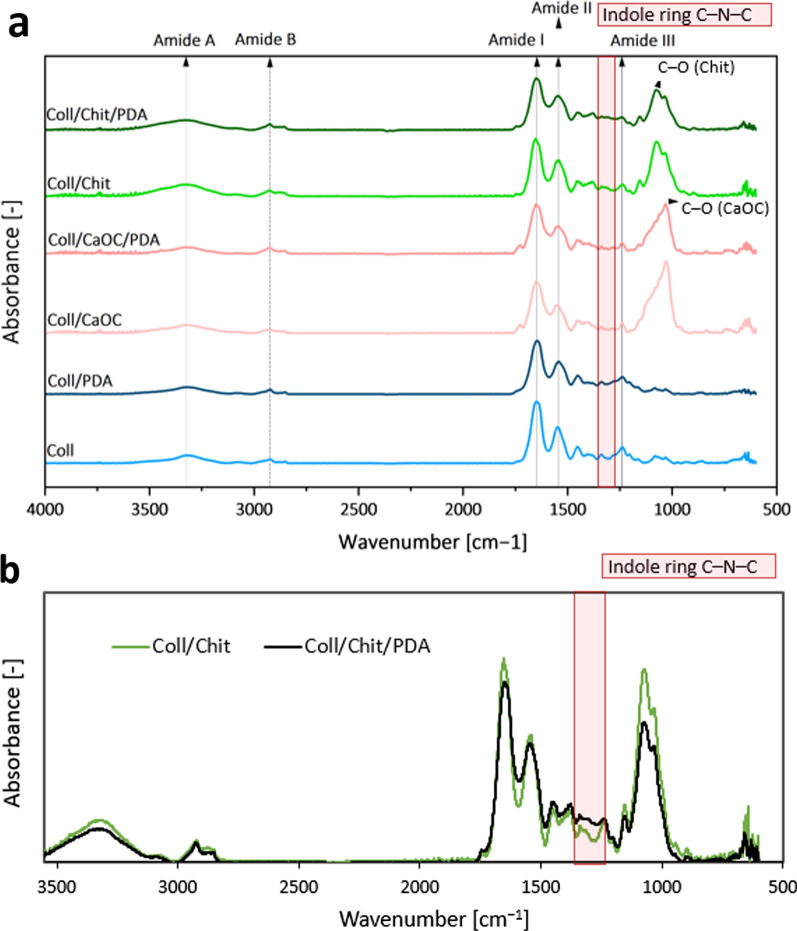
Absorption FTIR spectra of porous foam and porous foam coated with PDA **a**; a more detailed explanatory overlapping absorption FTIR spectra of Coll/Chit and Coll/Chit/PDA porous foam for a better explanation of PDA bonding **b**

Figure 6a shows the ATR-FTIR spectra of the PDA and non-PDA Coll, Coll/Chit and Coll/CaOC foamed samples, which consist of the characteristic absorptions related to the O–H group and the N–H stretching bonds including the typical collagen amide A at 3325 cm^−1^ and the amide B at 2924 cm^−1^. Amide I is attributed to the stretching vibrations of the C=O groups at 1600–1800 cm^−1^. The vibrations of the N–H bands and the vibrations of the C–N are associated with amide II (1470–1570 cm^−1^). The vibrations of the C–N stretching, the N–H bending, and the vibrations of the CH_3_ groups belong to amide III at 1250–1350 cm^−1^ [47]. Collagen amide bonds A, B, I, II, and III are confirmed in all types of PDA and non-PDA samples. The presented absorption spectra look very similar in the whole wavenumber range. It is clear that the amount of individual biopolymers (Coll, CaOC, Chit) is large compared to the amount of PDA coating layer, so that very often the bands of PDA are overlap with the bands of collagen. The addition of PDA has already been characterized by the C–O–H of the catechol groups of PDA at 1410 cm^−1^ and the indole rings visible at about 1350 cm^−1^, which has also been shown to depend on the concentration of PDA (from 0.5 to 10 mg.mL^−1^) [48–50]. The amount of PDA added (2 mg.mL^−1^) in our experiments and the washing process during the preparation of the samples led to a lower final concentration of PDA in the samples that just coated the biopolymer fibers with thin layer. Figure 6b shows in more detail representative spectra of the PDA and the non-PDA coated Coll/Chit sample with a highlighted band of the PDA indole ring in the region between 1230 and 1350 cm^−1^ assigned to the C–N–C stretching. The presence of Chit and CaOC are clearly seen as vibration of carbon–oxygen single bond, denoted as C–O at 1076 cm^−1^ and 1031 cm^−1^, respectively.

### PDA influences the mechanical behaviour of bilayers

**Fig. 7:**
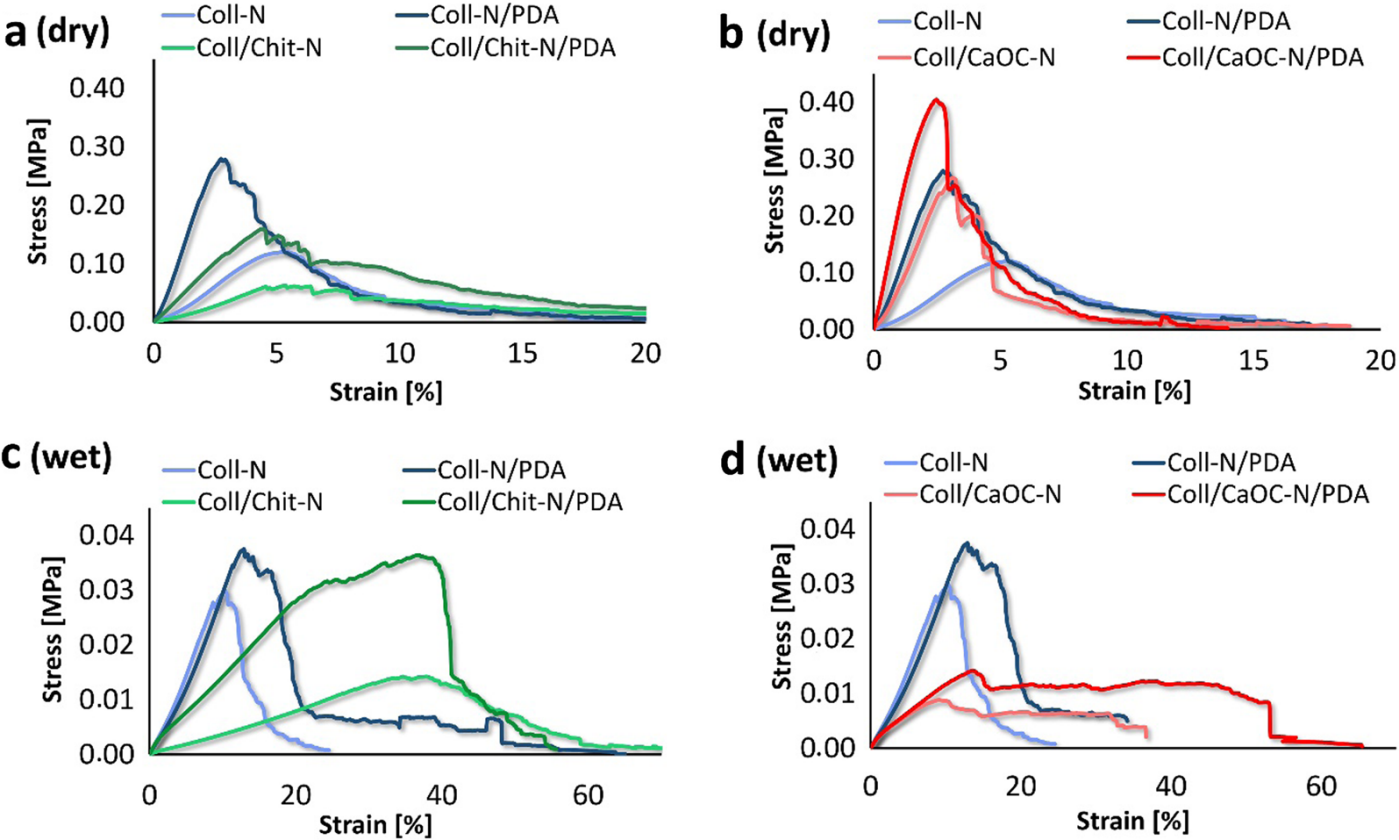
Stress–strain curves of cross-linked bilayers during both dry **a**, **b** and wet **c**, **d** measurements influenced by PDA addition. Collagen foam with cross-linked nanofibers is represented as Coll-N; collagen/chitosan foam with cross-linked nanofibers is represented as Coll/Chit-N; and collagen/oxidized cellulose foam with cross-linked nanofibers is represented as Coll/CaOC-N. The mark ‘PDA’ represents additional polydopamine coating on bilayer

The mechanical properties of the material are critical not only for cell responses, but also for handling during surgery. The following tensile curves (Fig. 7a–d) represent the stress–strain behavior of the cross-linked bilayers, and their representatives differ with the additional PDA coating. Non-cross-linked nanofibers were excluded from the measurement because of weak adhesion between the layers. Each tensile curve is characterized by a linear region with continuously increasing stress. The strain also increases and the material is in elastic deformation. In this region, the modulus of elasticity (the elastic modulus E) is constant and describes the stiffness of the material. Upon further increase in stress, the material transitioned from elastic deformation to yielding region, indicating the limit of elastic behavior and the onset of fiber failure. Eventually, each curve reached a point of ultimate tensile strength (UTS), the point representing the highest load that the material can handle. After UTS, the material is weakened, which is represented as a tearing phase. During the measurement in the dry state, the PDA stiffened the bilayer, and the tearing phase is faster than under hydrated conditions, which is seen mainly in the Coll/CaOC-N and Coll/CaOC-N/PDA bilayers in Fig. 7b and d. The tensile curves obtained during the measurement in the hydrated state (Fig. 7c and d) more clearly revealed a yielding region where the material reaches a certain degree of permanent destructiveness, observed mainly in the Coll/Chit-N and Coll/Chit-N/PDA bilayer. Under moisture conditions, similar to the conditions of a real environment, the elongation of the PDA-modified bilayer was greater than that of the cross-linked representatives, but with significantly lower applied UTS. The hydrated state allows the material to expand, and the retained water can act as a plasticizing agent. The elongation reached at least 50% of the original length, mainly for the Chit material. On the contrary, in the dry state, the addition of PDA reduced elasticity and the elongation of the PDA-modified bilayers was significantly lower than that of the non-PDA samples.

**Fig. 8:**
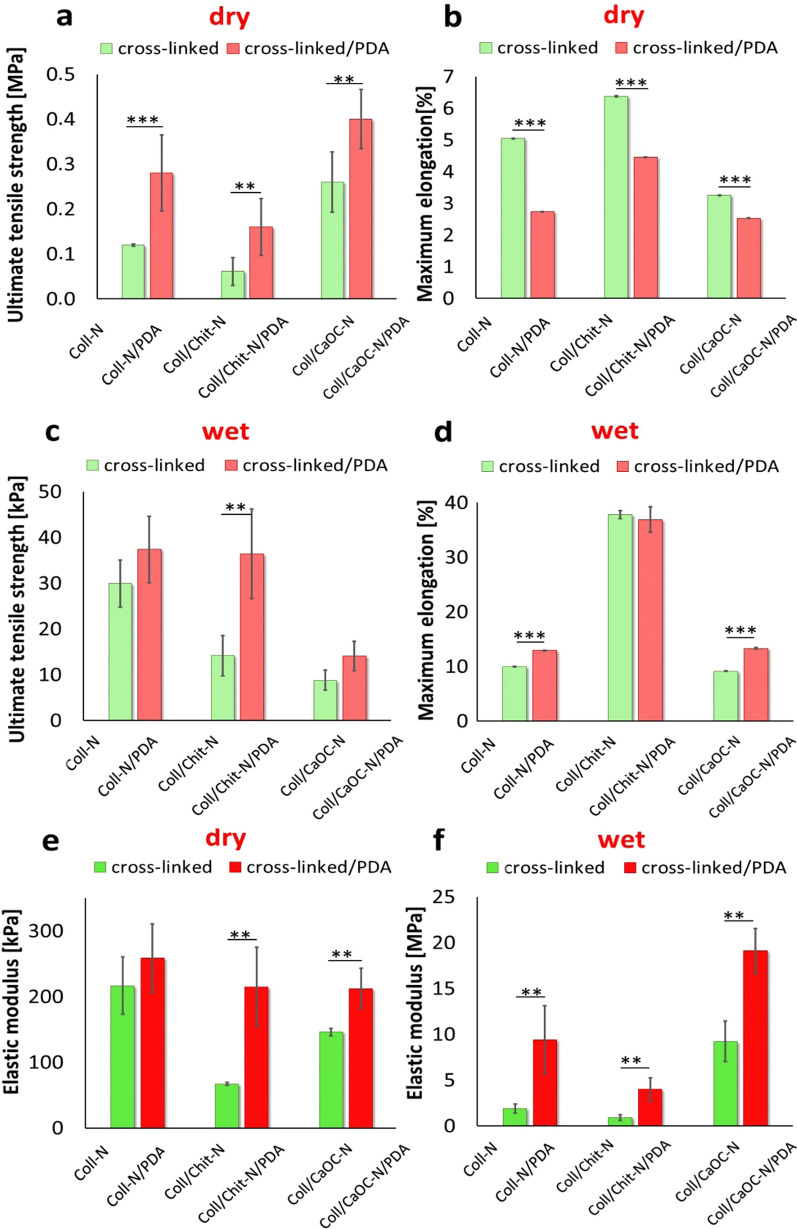
The summary of the ultimate tensile strength (UTS) values **a**, **c**; maximum elongations **b**, **d** and elastic modulus **e**, **f** measured in both dry and hydrated state. Collagen foam with cross-linked nanofibers is represented as Coll-N; collagen/chitosan foam with cross-linked nanofibers is represented as Coll/Chit-N; and collagen/oxidized cellulose foam with cross-linked nanofibers is represented as Coll/CaOC-N. The mark ‘PDA’ represents additional polydopamine coating on bilayers

All the values obtained for elongation, UTS, and the elastic moduli are depicted in Fig. 8. In general, PDA significantly affected the stiffness of each bilayer in both environments. Higher UTSs were measured in the presence of PDA, resulting in the highest mechanical resistance. The most resistant between the PDA bilayers was observed for the Coll/CaOC-N/PDA sample with UTS of 0.40 ± 0.07 MPa and maximum strain of 2.53 ± 0.01% followed by the Coll-N/PDA bilayer with UTS of 0.28 ± 0.09 MPa and strain of 2.74 ± 0.01% and finally the Coll/Chit-N/PDA bilayer with UTS of 0.16 ± 0.06 MPa and strain of 4.45 ± 0.01% (Fig. 8a and b). The hydrated state caused a decrease in stiffness; Coll/CaOC-N/PDA could maintain the structure at UTS of 14.09 ± 3.20 kPa and could achieve a maximum elongation of 13.30 ± 0.17%, Coll-N/PDA resisted at UTS of 37.34 ± 7.23 kPa, while achieving a maximum elongation of 12.9 ± 0.06%, Coll/Chit-N/PDA with UTS of 36.41 ± 9.82 kPa and with an elongation of 36.90 ± 2.34% (Fig. 8c and d). Figures 8e and fb represent the elastic moduli of all prepared bilayers. The modulus is orders of magnitude lower in the hydrated state with E ranging between 67 and 258 kPa. In the dry state, the E values of the bilayers are in the range of 0.7 MPa to 19.0 MPa. However, the addition of PDA significantly enhances the resistivity of material in the hydrated state.

### PDA influences the swelling behaviour and enzymatic degradation of the porous foam

**Fig. 9:**
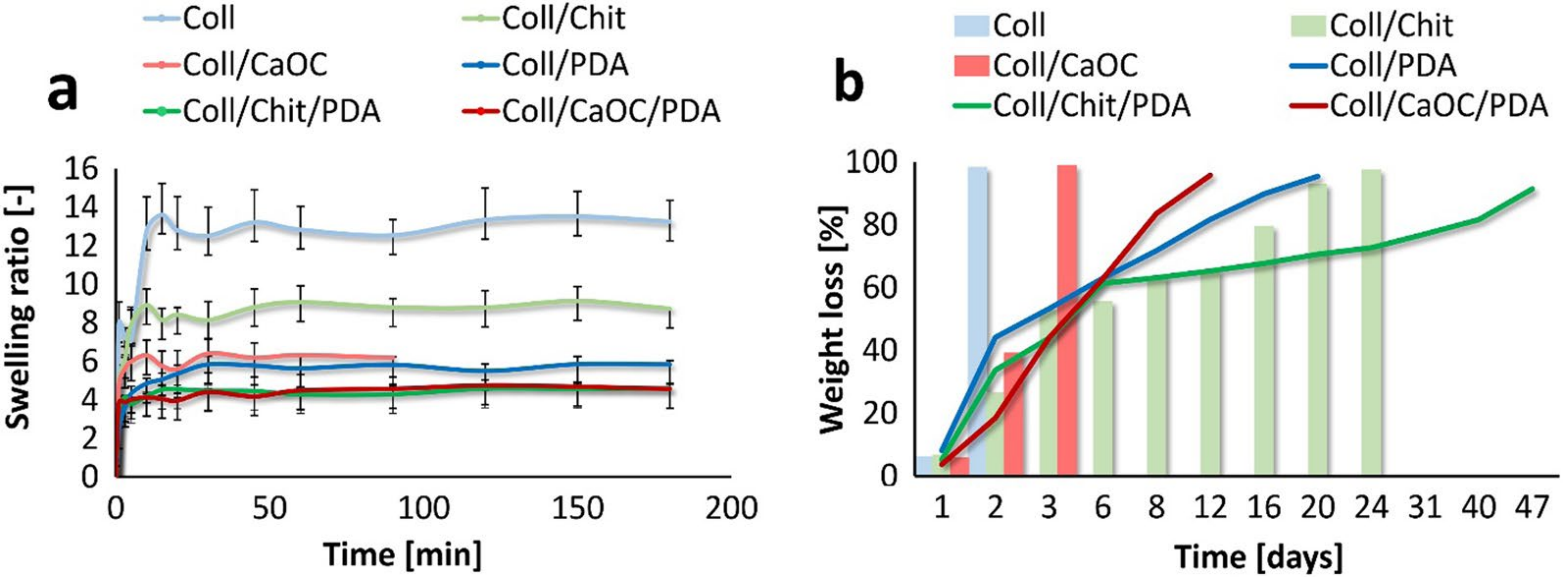
Swelling behavior **a** and enzymatic degradation **b** of porous foams. The collagen foam is Coll; collagen/chitosan foam is Coll/Chit and collagen/oxidized cellulose foam is Coll/CaOC. The mark ‘PDA’ represents additional polydopamine coating on porous foams

Figure 9 shows the swelling process and the enzymatic degradation of the porous foams. The highest swelling capacity was attributed to Coll, followed by Coll/Chit, and finally Coll/CaOC foam. In principle, Coll fibers have a good affinity to water and swell when water is absorbed. Once Coll is cross-linked with Chit, the swelling ratio decreases with the number of hydrophilic groups. CaOC with reduced hydrophilicity because of its calcium salt led to reduced water uptake. As shown in Fig. 9a, PDA significantly reduced the swelling ratios of all porous foams.

Enzymatic degradation is an important parameter to observe the stability of the designed scaffold in *in* *vitro* conditions. Degradation occurred in the enzymatic environment of collagenase, which is effective against Coll. The degradation process ended when sample handling was restricted. The addition of PDA significantly increased the lifetime of the Coll/PDA foam by 18 days, Coll/Chit/PDA needed 47 days and Coll/CaOC/PDA fully digested after only 9 days (Fig. 9b). Each scaffold achieved more than 95% weight loss at different times due to enzymatic degradation of Coll and hydrolytic degradation of polysaccharide.

### PDA influences the viability and proliferation of mouse fibroblasts in porous foam

**Fig. 10:**
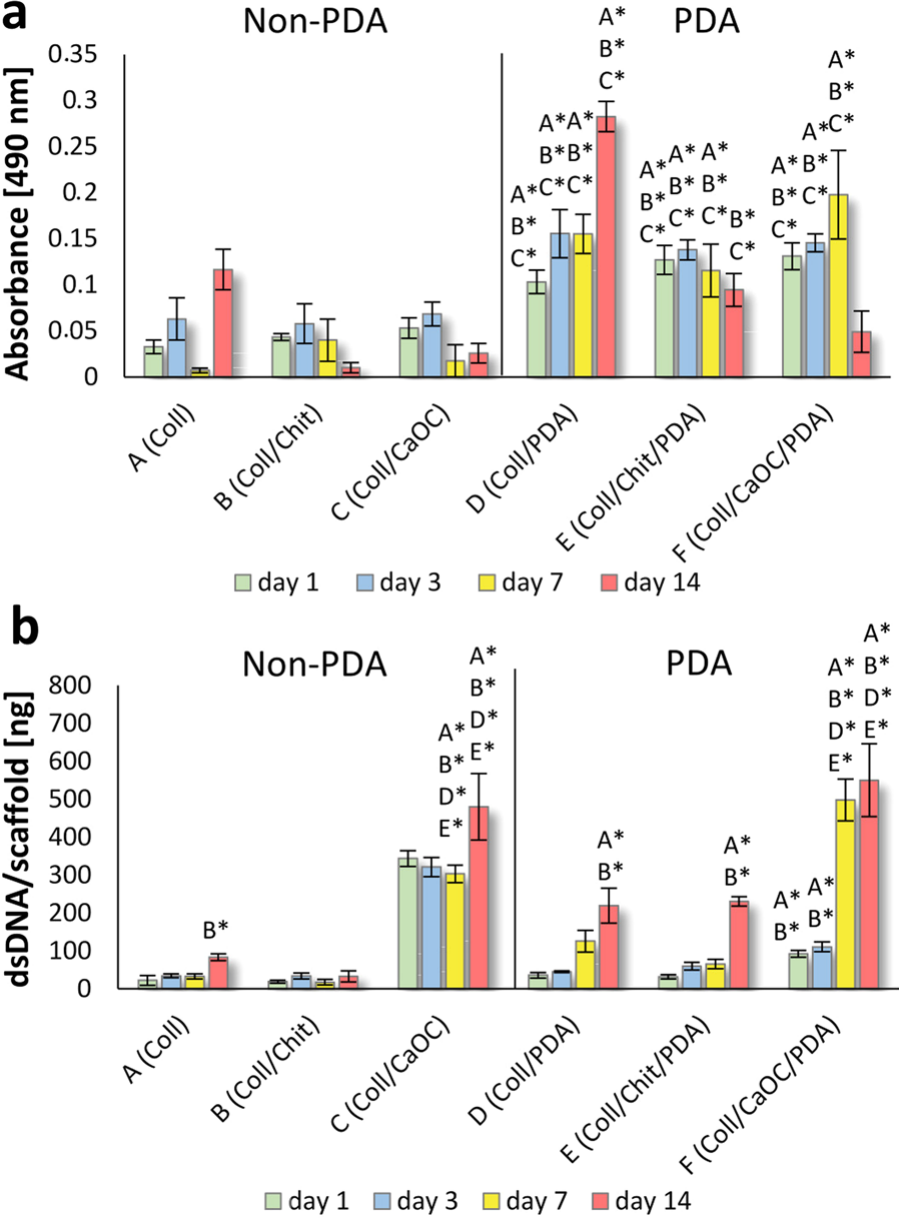
Metabolic activity **a** and proliferation **b** of murine fibroblasts cell line 3T3-A31 seeded on porous foams. The collagen foam is Coll; collagen/chitosan foam is Coll/Chit and collagen/oxidized cellulose foam is Coll/CaOC. The mark ‘PDA’ represents additional polydopamine coating on porous foams. Statistical significance marked with a group name (p < 0.05) and asterisk group (p < 0.05) and name of group with asterisk (p < 0.001), triangle sign for the highest value (p < 0.001)

Only porous layers were tested *in* *vitro*, because this layer is intended for the formation of fibroblasts and mainly to monitor the effect of the addition of PDA on cell processes. The metabolic assay showed that the highest sustained metabolic activity of the cultured cells (on day 14) was characterized in materials in the presence of PDA, Fig. 10a. Significantly more metabolically active cells were found in Coll/PDA compared to Coll/Chit/PDA and Coll/CaOC/PDA, respectively, on day 14. Cells in the Coll/CaOC/PDA sample proliferated strongly between day 7 and day 14 (Fig. 10b); therefore, they may have reached confluence associated with a decrease in metabolic activity (Fig. 10a). However, a good activity was demonstrated by the Coll/CaOC/PDA sample on day 7. As evident in Fig. 10b, the dsDNA quantification assay showed that the cell proliferation was remarkable in Coll/CaOC coated with PDA, with double-stranded DNA reaching 749 ng of dsDNA per scaffold on day 14 (Fig. 10b). PDA also significantly supported the proliferative activity of cells seeded on Coll and Coll/Chit scaffolds. In this case, the CaOC type of cellulose has shown very remarkable results even without the presence of PDA compared to that of Chit or Coll alone, probably due to the presence of calcium, essential element for cells.

**Fig. 11:**
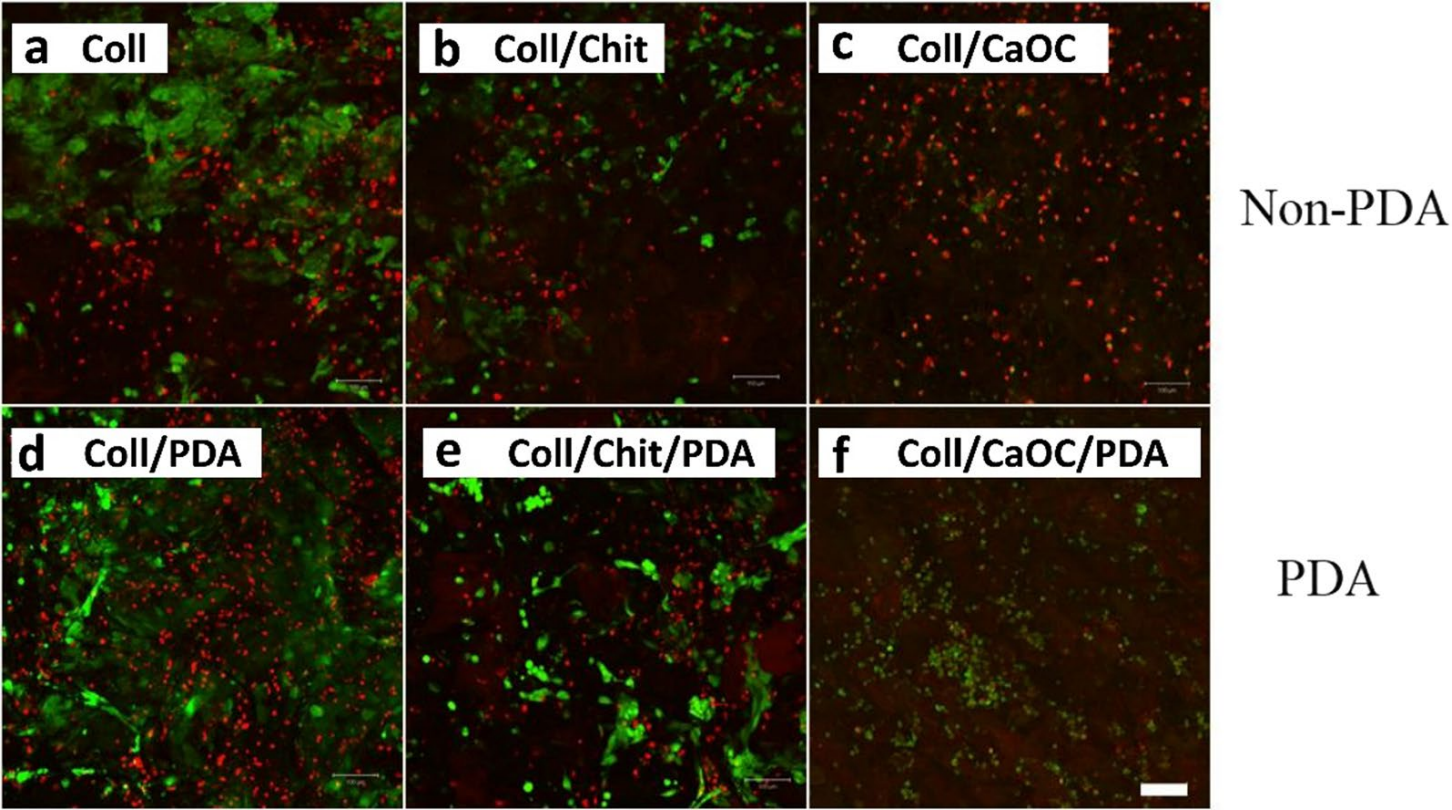
Live-Dead assay of murine fibroblast cell line 3T3-A31 seeded on porous foams on the 14th experimental day. Collagen foam is Coll; collagen/chitosan foam is Coll/Chit, and collagen/oxidized cellulose foam is Coll/CaOC. The mark ‘PDA’ represents an additional polydopamine coating on porous foams. The cytoplasm of living cells (green fluorescence) and dead cells (red fluorescence). Scale bar 100 µm, objective 10 ×

**Fig. 12:**
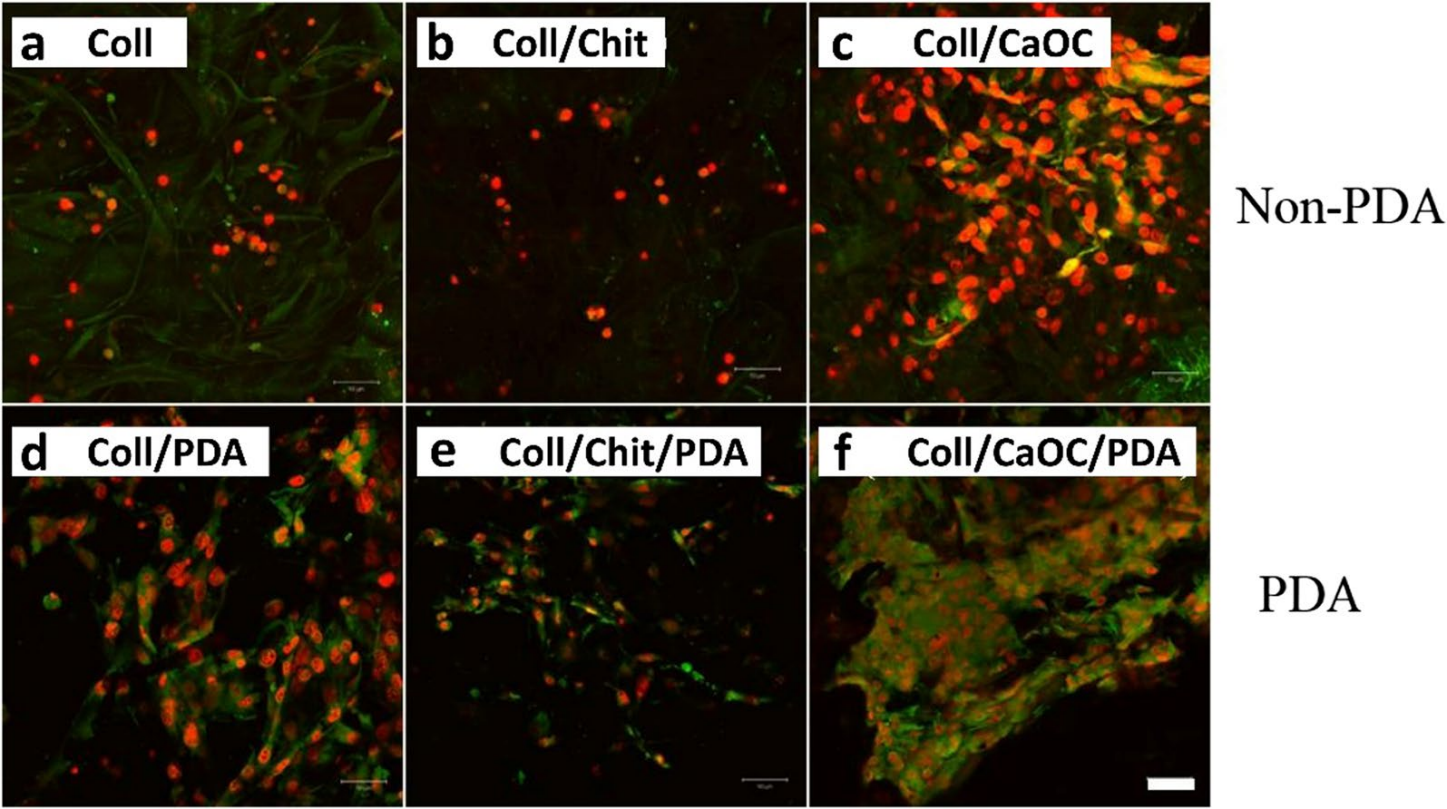
The cell distribution on the scaffolds—day 14. Collagen foam is Coll; collagen/chitosan foam is Coll/Chit, and collagen/oxidized cellulose foam is Coll/CaOC. The mark ‘PDA’ represents an additional polydopamine coating on porous foams. Cytoplasmic membranes (DiOC6[3], green signal), cell nuclei (propidium iodide, red signal). Scale bar 50 µm, objective 20 ×

**Fig. 13:**
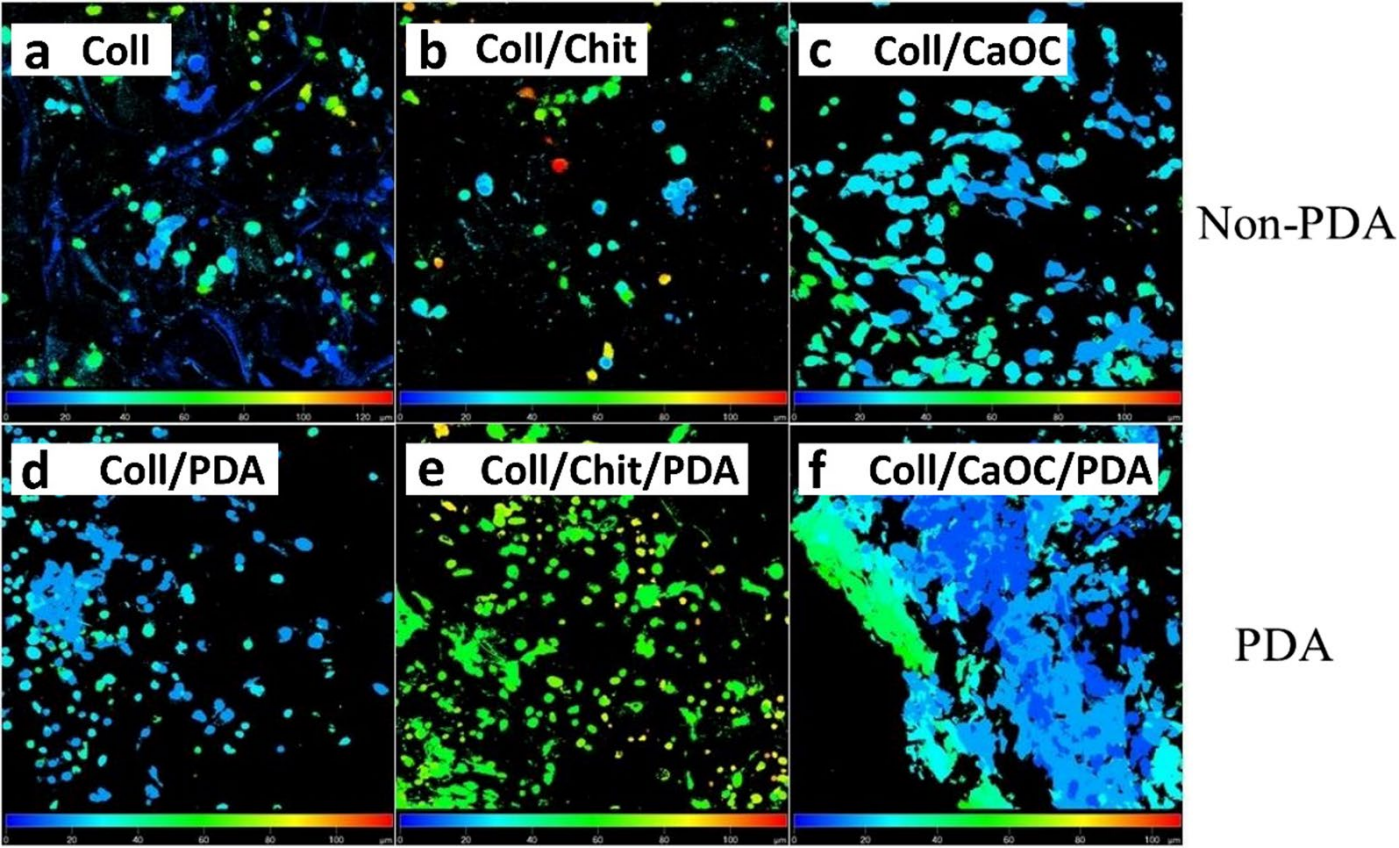
Color-coded depth projection based on the signal of cells stained with propidium iodide and DiOC6(3) on day 14. Collagen foam is Coll; collagen/chitosan foam is Coll/Chit, and collagen/oxidized cellulose foam is Coll/CaOC. The mark ‘PDA’ represents an additional polydopamine coating on porous foams. Depth view from 0 µm (blue) to 120 µm (red)

Fibroblast viability was performed by staining the cytoplasm of viable cells (expressed by green fluorescence) and the nuclei of nonviable cells (expressed by red fluorescence) (Fig. 11a–f). Propidium iodide was used since the cytoplasmic membrane of living cells is not permeable to it. Thus, living and dead cells can be distinguished. A significant difference was observed especially in the Coll/CaOC group without and with PDA. The PDA coating resulted in an increase in the number of viable cells. PDA also appears to promote the formation of cytoplasmic protrusions and their spread in PDA treated samples compared to the non-PDA treated samples (Fig. 12a–f). The cell cytoplasm surrounded the nucleus more closely in groups without PDA. PDA treatment in the Coll/CaOC scaffold appeared to promote the formation of prominent cytoplasmic structures. A color-coded projection of the cells evaluated from confocal microscopy has shown the cells in deeper layers (Fig. 13). The deeper layers are shown in green and yellow color and show that the fibroblasts were in the deepest layers present in the Coll/Chit foam coated with PDA (Fig. 13e). The cells were up to 60–80 µm deep. While in all other groups, they mostly migrated only around 20 µm deep. The higher amount of cells in deeper cell layers may arise from a larger pore size or open pores, and from better nutrition diffusion from the upper part of the scaffolds. This suggests that the scaffold is suitable for cell migration to deeper layers and allows sufficient medium flow to maintain cell viability.

### Wound healing of porcine skin with bilayers

**Fig. 14:**
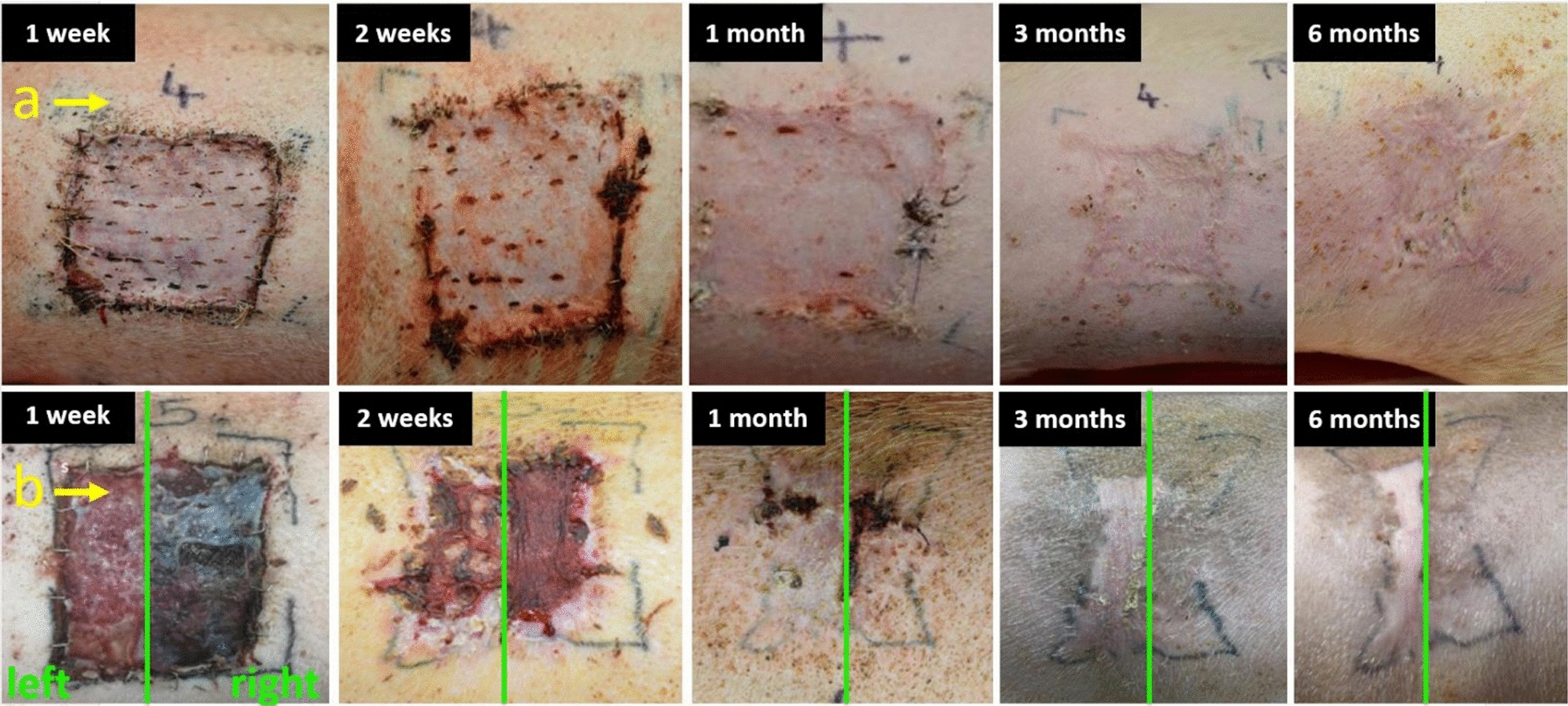
Closure of full-thickness wounds after implementation of control group—native porcine skin **a** and bilayer made of collagen/oxidized cellulose foam with cross-linked nanofibers—Coll/CaOC-N (b-left) and bilayer coated with polydopamine—Coll/CaOC-N/PDA (b-right) for 6 months

Coll/CaOC-N and Coll/CaOC-N/PDA bilayers were selectively chosen for in vivo experiments considering their *in* *vitro* results. Figure 14a. shows photographic monitoring of wound closure and contraction after application of control group, native porcine skin. Figure 14b shows the photographic monitoring of wound closure and contraction after application of bilayers. This wound was divided into two parts; on the left side, the Coll/CaOC-N bilayer was applied, and on the right side, the Coll/CaOC-N/PDA bilayer was applied. By month 6, the wounds had healed and contracted. On the right side, where the PDA-based bilayer was applied, hair growth was observed since month 3. Moreover, from visual observation, the resulting scar is more evident from the control group.

### PDA influences the expression of inflammatory cytokines during wound healing

**Fig. 15:**
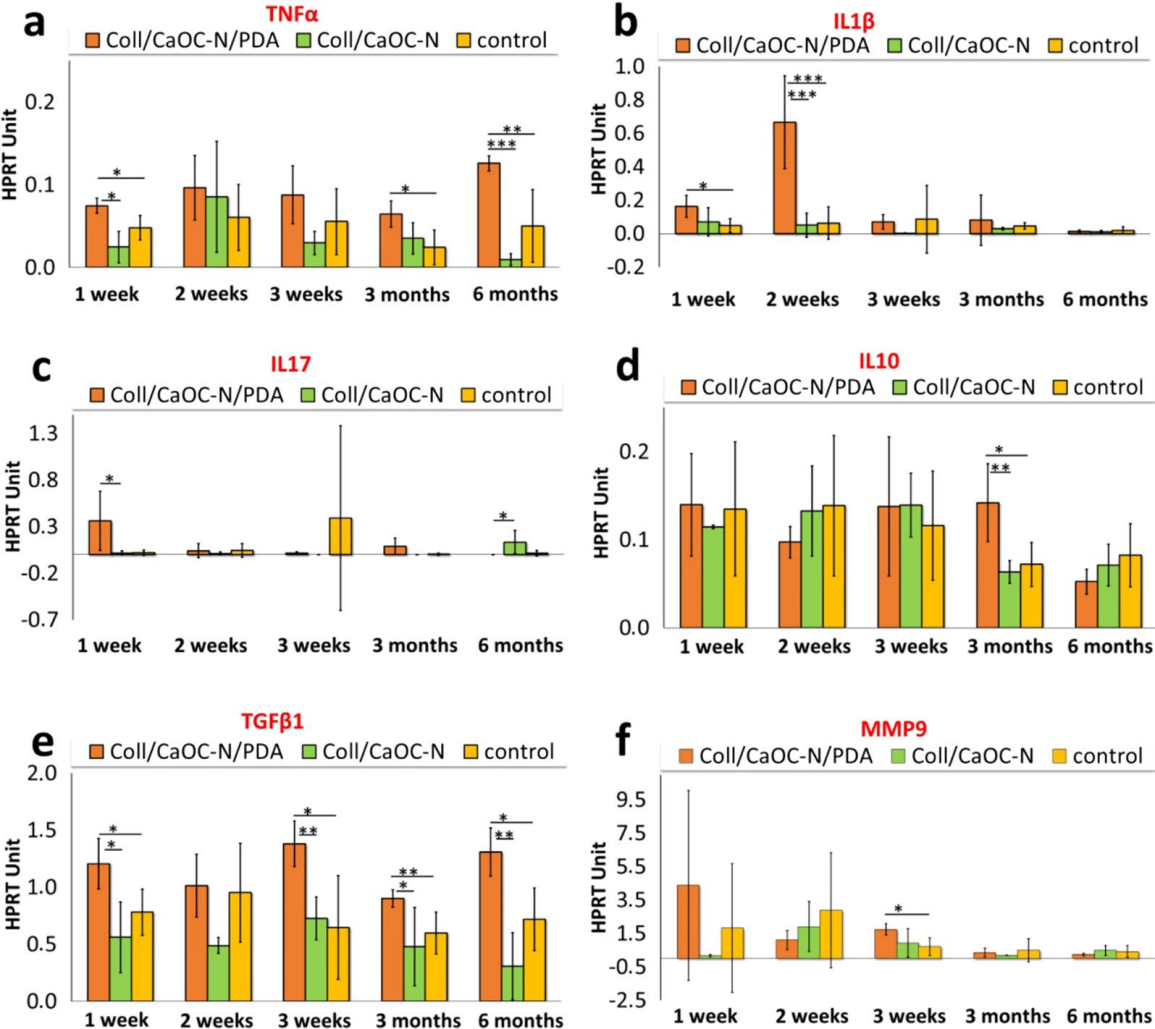
Time dependence of mean expression of the HRT unit of pro-inflammatory cytokines TNFα (**a**), IL1β (**b**), IL17 (**c**), MMP9 (**f**), anti-inflammatory cytokines IL10 (**d**) and growth factor TGFβ1 (**e**). Each panel represents a specific time of cytokines expression (1 week–6 months) and implanted material, where a bilayer made of collagen/oxidized cellulose cross-linked with nanofibers coated with polydopamine is Coll/CaOC-N/PDA (orange color panel), a bilayer made of collagen/oxidized cellulose cross-linked with nanofibers is Coll/CaOC-N (green color panel).( The panel of the control group (yellow color panel) is the native porcine skin. Statistical significance (*p < 0.05), (**p < 0.005), (***p < 0.001)

The expression of all cytokines in response to PDA treatment during the first three weeks after material transplantation, after month 3 and month 6 is shown in Fig. 15. The level of pro-inflammatory cytokine TNFα at week 1 was significantly higher for material involving PDA compared to sample without PDA or control (p < 0.05). Week 2 and week 3 did not show significance for TNFα when PDA was added. However, at the end of healing (month 3), the expression of TNFα was significantly higher for the sample treated with PDA compared to the control (p < 0.05). At month 6, TNFα significantly increased, again for the PDA sample, compared to the control and the non-PDA variant (p < 0.005 and p < 0.001). The expression of other pro-inflammatory cytokine IL1β for the PDA sample at week 1 was significantly higher that of the control (p < 0.05). At week 2, IL1β increased significantly for the PDA sample compared to the non-PDA and control (p < 0.001). This expression was then reduced and was comparable to that of the non-PDA material. Another pro-inflammatory cytokine IL17 was significantly higher at week 1 for the PDA material (p < 0.05), while month 6 showed a significant decrease in IL17 level (p < 0.05), compared to the non-PDA variant. In contrast, PDA was found to promote the elimination of inflammation, as it supported the expression of the anti-inflammatory molecule IL10 (month 3) during healing (p < 0.005). Another cytokine TGFβ1 (transforming growth factor) was found to be significantly expressed in the healing stages of the material with added PDA (besides week 2). The level of the last cytokine MMP9 (an enzyme of the matrix metalloproteinase (MMP) family) increased in the presence of PDA to a significantly higher level at week 3, compared to the control. Expressions of MMP9 in the rest stages of wound healing were comparable between materials and not significantly different.

**Fig. 16:**
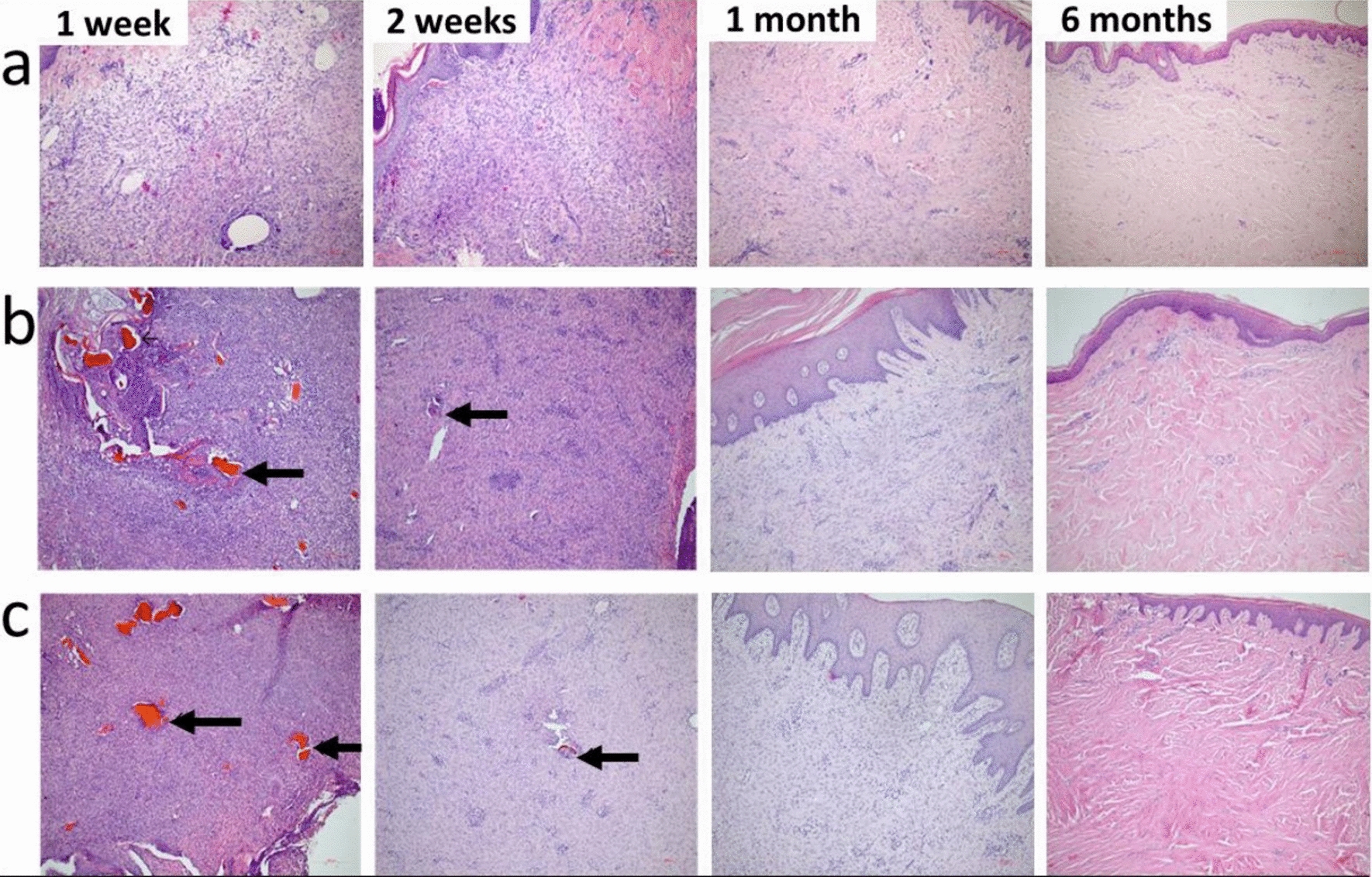
Histological sections at various times of tissue harvesting after implantation of the control group-native porcine skin **a**, a bilayer of collagen/oxidized cellulose cross-linked with nanofibers (Coll/CaOC-N) presented as a well-organized scar tissue **b**, collagen/oxidized cellulose cross-linked with nanofibers coated with polydopamine (Coll/CaOC-N/PDA) the presence of macrophages and fibroblast-like cells in the 1st and the 2nd week after application, collagen-rich tissue with low presence of fibroblast-like cells 6 months after application **c**. Sections were stained with H&E, photographs at 200 × magnification. Remnants of the nanofiber layers are depicted in a black arrow

An early inflammation is also evident in the histological pictures in Fig. 16, the first week after implantation of Coll/CaOC-N and Coll/CaOC-N/PDA bilayers compared to the control. Here, a superficial skin defect is evident and the dermis is inflammably cellular with the implanted material. After two weeks, there is still a skin defect on the surface and the cellular granulation tissue is rich in capillaries and fibroblastic tissue. The content of residual implanted material, multinucleated macrophages, and fibroblast-like cells is presented in the surroundings. After one month, there is a fibrous cellular scar with well-organized fibroblast formation and mild chronic inflammation without implant material. The surface defect is already re-epithelialized. 6 months of healing shows an organized fibrous scar with a lack of fibroblastic cells and capillaries in the dermis. On the wound surface is the epidermis of the usual structure presented.

## Discussion

To date, of all acellular scaffolds, only human acellular dermis products, such as clinically proven AlloDerm and DermaMatrix appear to be the best option in skin tissue [51, 52]. Implantation of acellular scaffolds with cell cultures of fibroblasts and/or keratinocytes is associated with enormous costs and difficult regulations [53]. Currently, an example of a cell-free extracellular matrix represents Integra, made of Coll and chondroitin-6-sulfate with a silicone backing [54]. This matrix has also been seeded with autologous fibroblasts and keratinocytes, but it is not yet commercially available. It is time-deficient, as it requires 3 to 4 weeks for cultivation. Similarly, 2 weeks of keratinocyte cultivation is required by similar material [55].

In this research, bilayer acellular scaffolds were fabricated and modified with PDA to enhance biomechanical and biological properties and to see the healing effects of porcine skin, as well as in vitro tests with murine fibroblasts. The nanofibrous layer functionally and structurally represents the BM, and the porous foam represents the dermis layer. Fabricated nanofibrous BM is characterized by small pores and a very thin thickness 0.2 mm, compared to the fabricated dermis layer, which is about 2 mm. Its main objective is to block bacteria and ensure that fibroblasts do not travel from the dermis part to the epidermis part, as well as to support adhesion to a possible epidermal autograft. Porous foam with larger pores and higher thickness ensures fibroblast adhesion, proliferation, nutrient support, and general filling of the wound bed. PDA is obtained by auto-polymerization of dopamine and has already shown some advanced effects on material properties in tissue engineering applications, mainly in terms of mechanical and cellular performance [56, 57]. In this work, the PDA improved the mechanical properties of all bilayers under both dry and hydrated conditions. PDA resulted in an increase in UTS of approximately 58%, 62%, and 35% for Coll-N/PDA, Coll/Chit-N/PDA, and Coll/CaOC-N/PDA bilayers, respectively. In the hydrated state, PDA enhances the firmness mainly in Coll/Chit-N/PDA (by 61%). The hydrated environment provided to the PDA-coated bilayers with higher viscoelasticity. One of the striking features of healthy skin is its ability to return to normal after being stretched. The hydrated state and PDA significantly supported fibers elongation, which was considered beneficial because the hydrated conditions mimic the real tissue environment. The Coll/Chit-N/PDA bilayer was stretched by more than 80% in the hydrated state compared to the dry state. Samples Coll-N/PDA and Coll/CaOC-N/PDA elongated by more than 79–81% under hydrated conditions, respectively. Adding PDA to an already existing network of collagen and polysaccharide enhanced the mechanical rigidity of the material. PDA contains abundant hydroxyl groups of catechol and active amino groups, which can easily penetrate the network and physically cross-link with residues of protein/cellulose functional groups and form an interpenetrated polymer network. This type of network shows significantly better mechanical properties than "ordinary" polymer networks [58–60].

PDA has also slowed the enzymatic degradation, while the swelling capacity and pore size were reduced. Although the pore structure was reduced, and the material became more rigid, the Coll/CaOC/PDA showed the highest proliferative activity between day 7 and day 14 and satisfactory metabolic activity during the experimental period. Oxidized cellulose foams Coll/CaOC and Coll/CaOC/PDA, respectively, revealed favorable properties for initial cell adhesion and proliferation with possible further formation of the confluent cell layer supported by PDA. Greater spread of the cytoplasm and supported cell viability were also observed in all samples after PDA coating. In our experiments, we used the static culture of the scaffolds. In the majority of 3D scaffolds cultivated under static conditions, dead cells are visible after long-term cultivation, probably due to slow medium diffusion. In addition, the physico-chemical properties of the scaffold surface or scaffold degradation may negatively alter cell adhesion, proliferation, and viability of the cells. In our samples with PDA, significantly (p < 0.05) increased metabolic activity of cells was observed. This is in good agreement with Paccelli et al. [61]. They reported an increased wettability of gellan gum hydrogel coated with PDA and subsequently increased cell adhesion, spreading, proliferation, and focal protein and cytoskeletal protein expression. Regarding the surface of the nanofibrous layer, PDA also changed its surface morphology and was able to create different topologies depending on the porous collagen/polysaccharide foam, on which the nanofibers were fabricated. For example, some untreated residuals of the slightly acidic CaOC of the Coll/CaOC foam could reduce the polymerization of dopamine and create a more homogeneous film of PDA compared to ball-like structures on the rest of nanofibers. This can further affect the diffusion of molecules.

In reconstructing skin tissue, it is essential to choose an appropriate wound in a preclinical animal model. Most in vivo studies, including bilayer dressings, are evaluated in small mammals, especially rats, mice, rabbits, or guinea pigs due to their cost and easy-to-handle processes [62–64]. Recently, in vivo studies were performed with dopamine-modified materials that have also been shown to heal wounds in these small mammals, including rats and mice [65–68]. To our knowledge, only a few studies have used the pig model, as a result of expensive studies and difficulties due to its size [69–71]. In addition, there are no studies that demonstrate the effect of dopamine on porcine skin. Furthermore, wound healing in the pig model differs from wound healing in small mammals [72, 73]. Many authors have already suggested that pigs should be the preferred animal model due to similarities between their epidermis and dermis with humans [74, 75]. In this work, a crucial question regarding PDA-coated bilayer material is its influence on porcine skin during wound healing, especially anti-inflammatory behavior, as it is still little known. The Coll/CaOC-N/PDA bilayer was selected for the experiment and was compared with its non-PDA variant Coll/CaOC-N and the control group (native porcine skin). PDA supports the early stages of inflammation, since the levels of the pro-inflammatory cytokines TNFα, IL1β, and IL17 were highly expressed. Pro-inflammatory cytokines are among the first factors that are produced in response to wounds, as they must participate in the inflammation phase of wound healing with a moderate immune response only [76]. Proper levels of pro-inflammatory cytokines prevent infection and accelerate normal wound healing [77]. The expression of the anti-inflammatory cytokine IL10 and the growth factor TGFβ1 is higher in the middle stages of wound healing, due to PDA. PDA also supported MMP9, which could help in angiogenesis and neovascularization processes in the middle stage of healing [78]. The expression of all cytokines in this study supported the idea that PDA triggers the early stages of inflammation (1–3 weeks) and prolongs inflammation at the end of healing (6 months). At this point, the overactivation of immune cells and their protease products could inhibit tissue formation.(1) An inflammatory reaction is a dynamic process with a reparation phase followed by a rebuilding phase associated with macrophages activation and the production of TGFβ1 as well as MMPs. So far, there has been no direct evidence for the role of polydopamine in macrophage activity. The main anti-inflammatory function of polydopamine is attributed to the ability to eliminate reactive oxygen species (ROS) [79]. The excessive amount of ROS produced by neutrophils at the wound site may destroy biological macromolecules, and thus it can cause a reduction of the production of anti-inflammatory molecules and contrary increase the production of pro-inflammatory cytokines. PDA is capable of capture electrons and scavenge reactive oxygen species (ROS) via its catechol groups, which reduce inflammation and promote tissue regeneration [80, 81]. Another potential mechanism is already proposed, based on PDA extracts, which may either act as a scavenger of ROS or can activate antioxidant protein HO-1 and thus inhibit the inflammatory response.[82] Based on the wound closure and contraction in Fig. 14 with wound recovery accompanied by hair growth, and less visual scarring from the PDA bilayer, it could be stated that the increased inflammation in the healing process had no effect on the speed of wound recovery. Although histological sections in Fig. 16 reveal minimal differences in histological skin tissue composition among native porcine skin, Coll/CaOC-N and Coll/CaOC-N/PDA, especially in the 6th month after implantation, the authors of the study find this favourable. According to histological sections, the maturation scar is advanced in Coll/CaOC-N and Coll/CaOC-N/PDA tissue samples compared to native porcine skin. The gross observations then favorize Coll/CaOC-N/PDA over Coll/CaOC-N samples, as mentioned above. Elgharably [83] used Coll gel to heal full-thickness excisional wounds of the porcine model and also showed a robust inflammatory response, which resolved in a timely manner followed by an improved proliferative phase, angiogenic result and post-wound tissue remodeling. Middelkoop et al. [84] studied Coll-based materials and synthetic materials in domestic pigs and pointed to the disadvantages of dressing treatments, which were not revealed in in vitro studies. Philandrianos et al. [85] showed that after implanted artificial dermal substitutes (Integra, ProDerm, Renoskin, Matriderm and Hyalomatrix) and the control group there was no differential effect on the contraction of full-thickness porcine wounds after 2 and 6 months of healing. Har-el et al. [86] conducted a similar study between an electrospun soy protein-based scaffold (SPS) and Tegaderm^®^ implemented in a full-thickness wound in the pig model. SPS exhibited better re-epithelialization. Importantly, it should be taken into account that studies demonstrating no risk of PDA degradation products are lacking. Jin et al. [82] showed that the PDA extracts were mainly composed of dopamine, quinine and PDA segments. These degradable products of PDA showed no cytotoxicity, which is in good agreement with our study.

## Conclusions

This study evaluated the effect of dopamine coating in a fully resorbable and acellular bilayer scaffold made of polysaccharides and collagen. The PDA has a unique position in the creation of biomechanically engineered materials as it significantly changes the strength of the bilayer and promotes stability and viscoelasticity due to its polymeric network interpenetration. This also influences swelling capacity and porosity, which is considered desirable and beneficial for restoring skin function, since dopamine also supports fibroblast viability and proliferation. This research contributes to the findings that dopamine may enhance the inflammatory phase at the beginning of wound healing in the domestic pig model and shows possible support in the expansion of growth factor and anti-inflammatory cytokines in the middle and later stages of wound healing. Dopamine shows no toxicity during the healing process, and therefore the dopamine-modified bilayer is suitable as implantable material. Despite the remarkable results of the in vitro experiments, the question remains whether the utility of dopamine in vivo is not overestimated, as it has been shown to be resemble to the control group. The future perspective on the utility of dopamine is mainly supported by its remarkable results based on in vitro experiments with cells, which substantially prove the potential of dopamine. However, its mechanism of action is still not completely discovered and even this study failed to clarify the mechanism of dopamine, as well as its degradable products and possible influence on the body. This action at the molecular level needs to be discovered before any application, which would also provide a better understanding of its future incorporation into biomaterials, thus obtaining more pronounced in vivo results.

## Data Availability

Without restrictions.

## Abbreviations

b.w: Body weight
CaOC: Calcium salt of oxidized cellulose
Chit: Chitosan
Coll: Collagen
Coll/CaOC: Collagen/oxidized cellulose foam
Coll/CaOC-N: Collagen/oxidized cellulose bilayer (foam + cross-linked nanofibers)
Coll/CaOC-N/PDA: PDA coated collagen/oxidized cellulose bilayer (foam + cross-linked nanofibers)
Coll/CaOC-NX: Collagen/oxidized cellulose bilayer (foam + non-cross-linked nanofibers)
Coll/CaOC/PDA: Collagen/oxidized cellulose foam coated with PDA
Coll/Chit: Collagen/chitosan foam
Coll/Chit-N: Collagen/chitosan bilayer (foam + cross-linked nanofibers)
Coll/Chit-N/PDA: PDA coated collagen/chitosan bilayer (foam + cross-linked nanofibers)
Coll/Chit-NX: Collagen/chitosan bilayer (foam + non-cross-linked nanofibers)
Coll/Chit/PDA: Collagen/chitosan foam coated with PDA
Coll-N: Collagen bilyer (foam + cross-linked nanofibers)
Coll-N/PD: PDA coated collagen bilyer (foam + cross-linked nanofibers)
Coll-NX: Collagen bilyer (foam + non-cross-linked nanofibers)
Coll/PDA: Collagen foam coated with PDA
DMEM: Dulbecco’s modified eagle medium
E. coli: Escherichia Coli
EDC/NHS: *N*-(3-Dimethylaminopropyl)-*N*´-ethylcarbodiimide hydrochloride/*N*-hydroxysuccinimide
FGF2: Fibroblast growth factor 2
Gel: Gelatin
i.m: Intramuscular
i.p: Intraperitoneal
i.v: Intravenous
MRSA: Methicillin-resistant* S. aureus*
PBS: Phosphate-buffered saline
PCL: Poly(ε-caprolactone)
PDA: Polydopamine
PEG: Poly(ethylene glycol)
PLA: Poly(lactic acid)
PLGA: Poly(lactic-co-glycolic acid)
PVA: Poly(vinyl alcohol)
RhVEGF: Recombinant human vascular endothelial growth factor
ROS: Reactive oxygen species
SEM: Scanning electron microscope
SEMIPE: SEM image pore extractor
SeNPs: Selenium nanoparticles
s.c.: Subcutaneous
